# The nutritional and health benefits of ready-to-eat-cereal consumption: an updated technical review of global evidence

**DOI:** 10.3389/fnut.2026.1778338

**Published:** 2026-06-17

**Authors:** Lisa M. Sanders, Vicki Swier, Alice Qian Hou, Madhavi Trivedi, Ellen S. Michalski

**Affiliations:** 1 Cornerstone Nutrition, LLC, Battle Creek, MI, United States; 2 Kellanova, Battle Creek, MI, United States

**Keywords:** breakfast, cardiovascular disease, diet quality, nutrient intake, ready-to-eat cereal, type 2 diabetes

## Abstract

**Introduction:**

Ready-to-eat cereal (RTEC) is increasingly adopted for breakfast around the globe. Evidence from prior reviews links RTEC intake to higher micronutrient and fiber consumption and improved diet quality, although concerns persist about sodium and added sugar content. This technical review updates the research landscape from 2015 to the present, with an emphasis on new research from around the globe.

**Methods:**

PubMed and Google Scholar were searched to identify human observational studies, intervention studies, and dietary and economic modeling studies published from 2015 to September 2025. Eligible studies examined RTEC intake in relation to diet quality, nutrient intake, affordability, or health outcomes in children and adults. Data was extracted and summarized in a narrative format.

**Results:**

More than 70 publications were identified across North America, Europe, Oceania, Asia, and Latin America, with few studies in Africa. Most studies were observational. Across countries, RTEC consumption was associated with higher intakes of vitamins, minerals, dietary fiber, and whole grains, and higher overall diet quality. Added sugar intake was similar between RTEC consumers and non-consumers, but total sugar was higher, in part due to co-consumption of milk and/or fruit. RTEC contributed minimally to sodium and saturated fat intake. Prospective cohort studies generally reported inverse relationships between RTEC, particularly those high in fiber and/or whole grain, and risk of chronic diseases and all-cause mortality. Emerging research areas include affordability and overall wellbeing.

**Conclusion:**

This updated review of the literature supports that RTEC consumption is associated with higher nutrient intakes and better diet quality around the globe. However, the literature remains dominated by data from high-income countries. As RTEC intake expands around the globe, more clinical trials and expanded dietary surveillance across countries are needed to help establish causal inference and clarify the role of RTEC in healthy diets around the world.

## 1 Introduction

Ready to eat cereal (RTEC) is one of the most widely consumed breakfast foods around the world. Traditionally considered a staple breakfast in the North America, Australia, and Europe, RTEC consumption has expanded globally with increasing adoption across Asia, Latin America, and Africa. This growth has been fueled by urbanization, increasing demand for convenience, and the recognition of RTEC as a potential contributor of micronutrients, whole grains, and dietary fiber (1, 2).

There has been considerable research into the relationship of RTEC consumption with health and nutrition outcomes, including associations of RTEC with better diet quality, nutrient intake, cardiovascular health, and body weight (3–10). While most evidence has been reported in cultures where RTEC is a traditional breakfast (7, 10–22), data also suggests similar findings in different cultures and dietary contexts around the world (23–26). RTEC consumption is also associated with higher intakes of certain chronically under-consumed food groups, such as whole grains, dairy and often fruit, compared to non-consumers of RTEC or those consuming other breakfasts (7, 10). These patterns may reflect both the contribution of RTEC to the diet as well as its inclusion in a broader healthy dietary pattern.

However, concerns related to RTEC consumption have focused primarily on added sugar and sodium content. While most studies report similar added sugar intake in RTEC consumers and non-consumers, total sugar intake is typically higher in RTEC consumers compared to breakfast skippers and some other breakfasts (7, 10, 14, 21, 22). This may be due to RTEC consumption in combination with other sugar containing foods, such as fruit and milk. Alternatively, RTEC has been reported to be a minor contributor to sodium in the diet (3, 10, 27).

Two seminal reviews on RTEC and health were published in 2014 and 2016 (1, 2). However, a considerable amount of evidence has been published since that time. A review on RTEC as it relates to non-communicable disease prevention was also recently published and includes data primarily from western countries (28). Therefore, the purpose of this current review is to provide an updated analysis of the research landscape for RTEC, including new research on previously established and emerging outcomes from 2015 to the present with an emphasis on research across different parts of the world (4, 6, 8, 23, 24, 26).

## 2 Materials and methods

A comprehensive literature search was conducted using PubMed and Google Scholar to identify studies examining the intake of ready to eat cereals on human health and nutrition outcomes. The search strategy was intentionally designed to prioritize sensitivity over specificity to capture the full scope of the literature, including emerging health outcomes. For this reason, broad exposure terms (“ready-to-eat cereal” OR “breakfast cereal” OR RTEC) were used without restricting the search to predefined outcomes. Given this broad approach, the review should be interpreted as a narrative synthesis rather than a systematic review. The search was limited to English language publications from 2015 to January 2025. Another search was performed in September 2025 to identify any additional papers published since the previous search. Reference lists of included papers and systematic reviews or meta-analyses were reviewed for publications not identified in the database searches. Titles and abstracts were reviewed for inclusion followed by a confirmatory review of the full text.

Studies were included if they met the following criteria:

Population: human studies in adults or children

Intervention/Exposure: consumption of RTEC

Outcomes: nutrient intake, diet quality, health outcomes, affordability, or related dietary or economic measures

Study design: observational studies, intervention trials, dietary modeling studies, cost, or affordability analyses

Studies were excluded if they included cereal as a delivery vehicle for another intervention (e.g., cereal with an added novel ingredient), or if the research examined topics unrelated to consumption and dietary or health outcomes (e.g., marketing, labeling studies). Animal, *in vitro*, or any studies published prior to 2015 were also excluded. Intervention studies on hot cereal only were excluded; however, observational studies did not always report if the RTEC or breakfast cereal exposure included hot cereals. When this distinction was unclear, studies were included and classified according to the definition used by the original authors.

Any publications that were unclear regarding eligibility were discussed among the authors to determine inclusion or exclusion. Data from each eligible study was extracted using a modified PICO (population, intervention/exposure, comparator, outcome) chart that also included study design, location, and any covariate adjustments (observational only). When multiple statistical models were reported within a study, data from the fully adjusted model were used for data extraction and synthesis. In observational studies, different classification systems were used to define “RTEC consumers” including frequency of consumption (high vs. low), amount consumed daily or weekly, and for cross-sectional studies, if RTEC was reported in a 24-h recall. To preserve the integrity of the original analyses, we utilized the exposure definitions in individual papers to classify RTEC consumers and compare them to non-consumers or less frequent consumers of RTEC. Details on how each study defined RTEC consumers is in Supplementary Table 1.

Given the heterogeneity in study design, populations, exposure, and outcomes, a structured narrative synthesis was conducted. Studies were grouped according to outcomes and findings were compared across study design, population characteristics, geographic context, and exposure/intervention. The synthesis focused on identifying patterns, consistencies and variability in the findings and factors that may have influenced associations. No formal risk of bias assessment of study quality scoring was performed.

## 3 Results

Since 2015, more than 70 publications examining RTEC and nutrient intake, diet quality, affordability, and health outcomes have been published. A summary of study characteristics and findings is provided in Supplementary Table 1. Most of these studies evaluate nutrient intake and diet quality (3–27, 29–53) or health outcomes (9, 12–14, 19, 24, 26, 35, 38, 40, 47, 54–73) with a few evaluating affordability (20, 21, 48, 74). Observational studies also dominate the literature with only a handful of intervention studies identified (35, 47, 62, 70, 71, 73). Studies have been conducted in a wide range of countries around the globe including Australia (11–14, 30, 33, 50, 61), Canada (8, 9, 20, 54, 65), Chile (24, 49), across Europe (18, 38, 52, 57), France (36), Ireland (32, 34, 37, 53), India (35), South Korea (26), Malaysia (25, 39, 67), Mexico (23, 55, 64), Spain (27), UK (22, 29, 47, 66, 69, 72, 73), and the US (3, 7, 10, 15–17, 21, 22, 31, 40–46, 48, 51, 56, 58–60, 62, 63, 68).

Across studies, RTEC was generally evaluated as a single exposure category; however, where data were available, findings for specific subtypes, such as whole grain or higher-fiber RTEC, are reported.

### 3.1 Nutrient intake

#### 3.1.1 Micronutrients

Across multiple countries around the world, consumption of RTEC is consistently associated with higher intakes of vitamins & minerals compared to non-consumption or lower consumption of RTEC. RTEC consumers generally report higher overall intake of several vitamins and minerals (Table 1) and are more likely to meet certain micronutrient requirements for the day compared to non-consumers (Table 2). These observations may reflect both the nutrient contribution of RTEC as well as a broader healthy dietary pattern among RTEC consumers.

**Table 1:** Publications since 2015 reporting higher daily intake of vitamins and minerals in RTEC consumers compared to those who consume less or no RTEC.

Vitamin or mineral	Reference
Vitamin A	Chile (24), France (36), Malaysia (25), US (3, 7, 10, 21, 40, 41)
B vitamins	Australia (12–14), Europe (38), Canada (9, 20), France (36), Malaysia (39), Mexico (23), South Korea (26), UK (22), US (7, 10, 21, 40, 41)
Vitamin C	Chile (24), Europe (38), France (36), Malaysia (25), UK (22), US (7, 21, 41)
Vitamin D	Canada (20), Europe (38), Ireland (32), Mexico (23), UK (22, 29), US (7, 10, 21, 22, 31, 40, 41)
Calcium	Australia (12), Canada (20), Chile (24), Europe (18, 38), France (36), Malaysia (25, 39), Mexico (23), South Korea (26), UK (22), US (7, 10, 21, 40, 41)
Iron	Australia (12), Canada (9, 20), Chile (24), Europe (38), France (36), Ireland (37), Malaysia (25, 39), Mexico (23), UK (22), US (7, 10, 15, 21, 40, 44)
Magnesium	Europe (18), UK (22), US (7, 10, 15, 21, 40, 41)
Potassium	Europe (18, 38), France (36), UK (22), US (7, 21, 40, 41)
Zinc	Mexico (23), Chile (24), US (7, 10, 21)

**Table 2:** Percentage of RTEC consumers and RTEC non-consumers not meeting nutrient adequacy for select vitamins and minerals.

Micro-nutrient	Children								Adults			
	Australia *% below EAR/AI*		Malaysia *% below 80% RNI*		South Korea *% below EAR/AI*		US *% below EAR/AI*		South Korea *% below EAR/AI*		US *% below EAR/AI*	
	RTEC consumers	Non-consumers	RTEC consumers	Non-consumers	RTEC consumers	Non-consumers	RTEC consumers	Non-consumers	RTEC consumers	Non-consumers	RTEC consumers	Non-consumers
Calcium	**25.6**	51.1	**37.6**	64.9	69.3	75.9	**29.4**	53.7	**9.5**	14.6	**27**	50
Folate	–	–	–	–	–	–	**0**	8.6	–	–	**0**	21
Iron	**1.8**	11.3	0	0.8	**31.3**	43.9	0	3.8	**22.3**	22.8	**0**	5
Niacin	–	–	**11.0**	31.0	**14.3**	26.5	0.1	0.7	**11.5**	–	**0**	3
Riboflavin	–	–	**2.3**	13.1	**8.4**	34.9	0.1	2.5	**20.3**	51.1	**0**	5
Thiamin	–	–	**16.2**	45.9	**8.7**	23.2	0.1	3.1	**15.4**	32.0	**0**	11
Vitamin A	–	–	6.4	9.0	**19.5**	38.6	**3.4**	35.4	**18.2**	37.8	**9**	56
Vitamin B12	–	–	–	–	–	–	0	2.6	–	–	**0**	8
Vitamin B6	–	–	–	–	–	–	0.1	2.8	–	–	**1**	19
Vitamin D	–	–	–	–	–	–	**84.6**	97.8	–	–	**83**	98
Zinc	–	–	–	–	–	–	**2.2**	11.1	–	–	**3**	21

Values in bold are significantly different from RTEC non-consumers in same country and age group. From (3, 10, 12, 26, 39).

**Figure 1:**
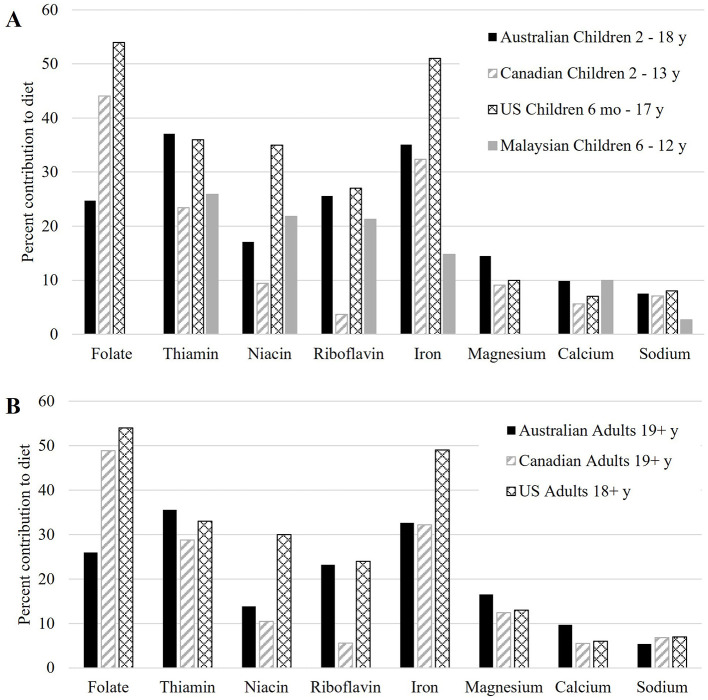
Contribution of RTEC to daily intake of several nutrients in children **(A)** and adults **(B)** around the globe. Data sourced from (3, 9, 10, 12, 13, 39).

In cross-sectional dietary analyses, RTEC contributes ≥10% of daily intake for several essential nutrients, including folate, iron, niacin, thiamin, riboflavin, and magnesium, across several countries (3, 10) (Figure 1). In the UK, RTEC provides ≥20% of daily intake of iron, folate, vitamin D, riboflavin, and thiamin for adults and children consuming RTEC (22). RTEC is not a major contributor to daily intake of calcium, contributing ≤ 10% of the daily intake in most countries. However, RTEC is frequently consumed with milk, a rich source of calcium. While these are population level findings, the impact of RTEC on nutrient intake at the individual level will likely vary by the type of RTEC and fortification.

Many of the micronutrients in RTEC result from fortification, which plays a critical role in improving nutrient intake and reducing the prevalence of micronutrient inadequacies in populations globally (29, 31, 32). A recent assessment of consumer scanner data in the US reported at least 77% of RTEC in the market are fortified with iron, vitamin A, vitamin C, or calcium (75). However, the prevalence of RTEC fortification in other countries has not been well investigated. A modeling study using NHANES data in the US reported that without RTEC fortification, the prevalence of nutrient inadequacies would markedly increase across multiple micronutrients (31). Additionally, fortification can be optimized to ensure a greater proportion of individuals meeting requirements without exceeding upper intake limits (76). A UK study demonstrated that fortification of RTEC with vitamin D (4.2 μg / 100 g RTEC) could significantly improve vitamin D status in both children and the elderly (29). Rehm, et al. (48), also reported that replacement of observed breakfasts in the US with RTEC could increase iron and folate intake by >50% while reducing intake of solid fats by >10%. However, a modest increase in added sugar (~1 teaspoon/d) may also be observed based on the models. Modeling studies are useful for understanding how changes in foods or diet patterns may impact nutrient intake, but they are based on assumed changes and should be interpreted with caution.

Intervention studies evaluating micronutrient intake were limited but generally consistent with observational findings (35, 47). In one 12-week intervention, girls aged 16–19 consumed either fortified or unfortified cereal with 150 ml low-fat milk daily (47). While both fortified and unfortified cereals increased dietary intakes of thiamin, riboflavin, and vitamin B6, the fortified cereal also enhanced intakes of vitamin B12, folate, iron, and vitamin D. Moreover, consumption of the fortified cereal significantly improved biomarkers of vitamin status for riboflavin, vitamin B12, folate, and iron, providing more direct evidence of its efficacy than intake data alone. Another 2-week study with fortified RTEC demonstrated an increase in intakes of vitamin B6, B12, and niacin (35).

#### 3.1.2 Dietary fiber

**Figure 2:**
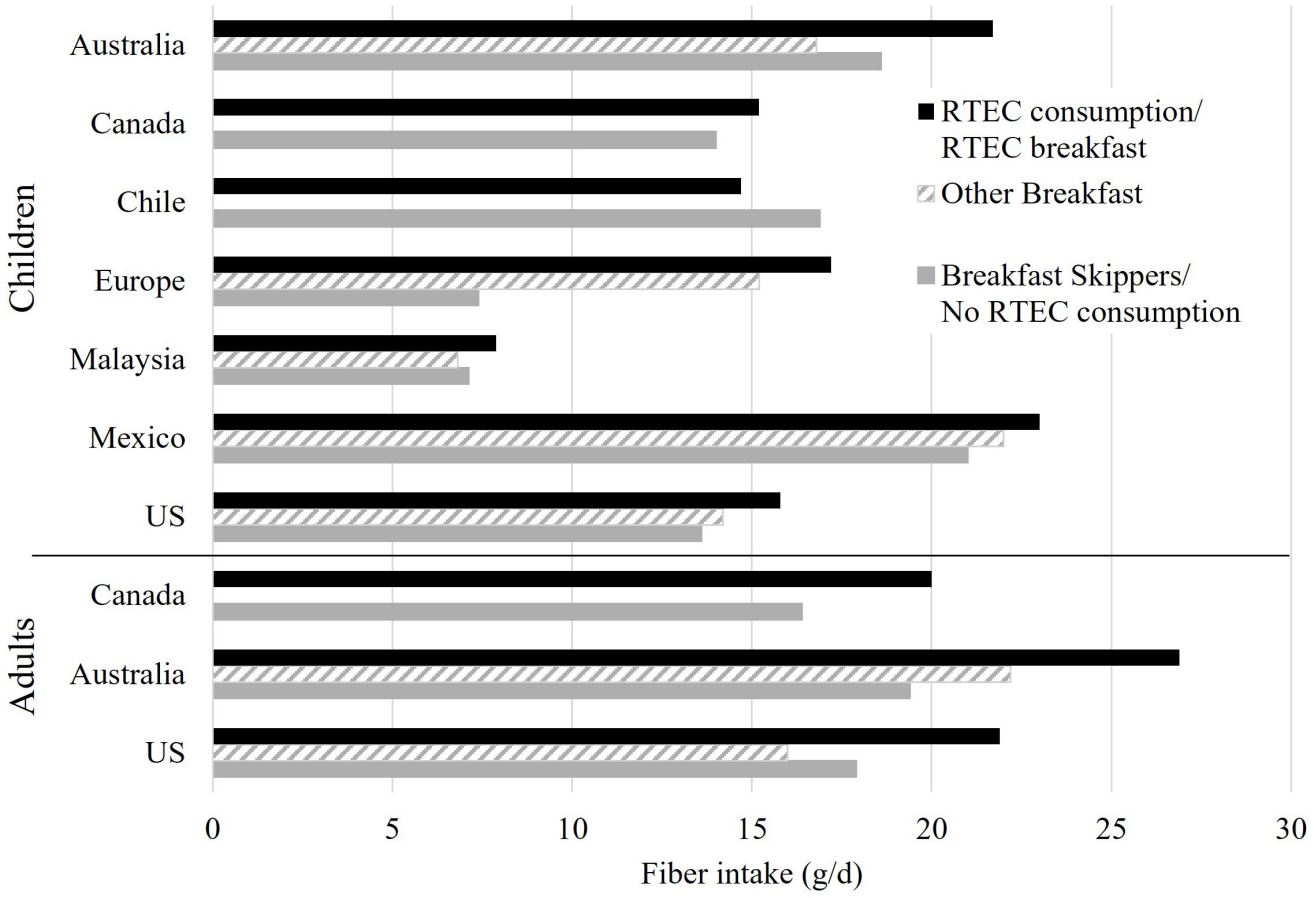
Daily dietary fiber intake for RTEC consumers (breakfast or any time of day), other breakfast consumers, and RTEC non-consumers (breakfast skippers or no RTEC at any time of the day), Data sourced from (9, 13, 14, 18, 20, 23–25).

Across studies conducted in Australia, Canada, Europe, Ireland, Malaysia, Mexico, and the US RTEC consumption is consistently associated with higher dietary fiber intake compared to not consuming RTEC or skipping breakfast (7, 10–23, 25) (Figure 2). For example, Australian children (2–18 y) consuming RTEC for breakfast, fiber intake was 15% higher compared to those consuming non-cereal breakfasts, and these children were 1.6 times more likely to meet the adequate intake for fiber (12). Similarly, Malaysian children consuming RTEC for breakfast had 15% higher fiber intake compared to those consuming other breakfast types (25). These observations may reflect both the contribution of RTEC to dietary fiber intake as well as an overall healthy dietary pattern among RTEC consumers.

Several studies also support RTEC as a meaningful contributor to daily fiber intake across populations, with the magnitude varying by intake level. Among Australian children in the highest quartile of fiber intake (29.7 g/day), RTEC contributed more than 10% of daily fiber and was the third-highest fiber source. For children in the lowest quartile of fiber intake (9.6 g/day), RTEC still contributed 6.1% and ranked as the fourth-highest source (11). Similarly, for Australian adults, RTEC was the number one fiber source among those in the highest quartile of fiber intake (39.6 g/day), contributing over 10% of daily fiber (11). Among adults in the lowest quartile (10.2 g/day), RTEC contributed 4.5% of daily fiber and was the fourth-highest source. In contrast, a study of adolescents across eight European cities reported RTEC contributed only 3.7% of daily dietary fiber intake, suggesting possible differences in dietary patterns and cereal consumption among adolescents (52). In Ireland, where RTEC intake is particularly high, RTEC provides approximately 10% of daily intake of dietary fiber which is the fifth-highest source. In Canada, RTEC provides more than 10% of daily fiber for children and over 20% for adults (9, 20). In the US, RTEC contributes approximately 20% of daily fiber intake for both children and adults (3, 10, 21) and an even greater contribution (>20%) in adults meeting fiber recommendations (16). A US modeling study estimated that replacing non-RTEC breakfasts with RTEC + milk could increase fiber intake across the population by 7.1%, although most individuals would still fail to meet the AI for fiber (48).

The contribution of RTEC to dietary fiber intake also depends on product composition. For example, a study in Chilean children reported no difference in fiber intake between RTEC consumers and non-consumers, which was attributed to the selection of lower fiber RTEC by the children (24). In contrast, data from Ireland indicated a shift in schoolchildren's cereals consumption patterns between 2003 and 2004 to 2017–2018, with an increase in high-fiber cereal intake (>6 g/100 g) and a corresponding decrease in low-fiber cereal intake (34). The authors note this shift was likely a result of the reformulation of RTEC to include more dietary fiber rather than behavioral changes in the child population because a comparison of the two surveys showed several RTEC varieties changed categories from low fiber (< 6 g/100 g) in 2003/2004 to high fiber in 2017/2018. Similarly, a product assessment of Australian RTEC products in 2020 revealed that 84% contained at least a source of fiber (≥2 g/serving), and 45% were classified as a good or excellent source (≥7 g/serving), (30). Additionally, fiber intake was the same in consumers of presweetened and non-presweetened cereals (14), suggesting differences in fiber are more influenced by product formulation than sugar content alone. Despite the contribution of RTEC to dietary fiber, overall intakes often remain below recommended levels (~21 g for children and 25 g for adults) in many populations (77).

#### 3.1.3 Added sugars

Evidence from observational studies generally report added sugar intake in RTEC consumers is similar or lower than non-RTEC consumers or breakfast skippers. In Australia, the UK, and the US, children and adolescents consuming RTEC for breakfast had similar daily added sugar intake compared to non-RTEC breakfast consumers and breakfast skippers (14, 21, 22). Furthermore, there was no difference in added sugar intake between those consuming presweetened RTEC (≥ 6 g/serving) and minimally or non-presweetened RTEC (< 6 g/serving), suggesting in the importance of the overall dietary composition when assessing added sugar intake (14, 40). Alternatively, in Mexico, children consuming RTEC for breakfast had higher daily added sugar intake compared to breakfast skippers indicating variability across populations and dietary context (23). In a slight difference from children, studies in adult RTEC consumers have reported lower daily added sugar intake compared to non-RTEC breakfast consumers and breakfast skippers (10, 13, 21). This may reflect changes in the added sugar content at breakfast in adults or a healthier dietary pattern among RTEC consumers. Studies assessing sugar content primarily used binary thresholds (e.g., ≥6 g/serving, < 6 g/serving) rather than a gradient of added sugar intake, limiting the ability to examine dose-response relationships.

As a portion of the daily contribution to added sugar intake, RTEC typically contributes less than 20%. In population-level analyses from the US and Ireland, RTEC contributes ≤ 6% of daily added sugars for both children and adults (10, 44, 53). However, among RTEC consumers, the contribution is 16%−18% of daily added sugars in the US (3, 10). Among children and adolescents consuming more than 15% of their daily calories from added sugars, RTEC contributes ~5% (~22 kcal/day) of this intake, with sweetened beverages, baked goods, and candy contributing 43, 15, and 9%, respectively. (46). Similarly, in Australia, RTEC accounts for ~12% of daily added sugar intake among child and adolescent RTEC consumers (14).

#### 3.1.4 Total sugars

Unlike added sugars, observational studies generally report higher total sugar intake in RTEC consumers compared to non-consumers and breakfast skippers (7, 10, 12–14, 20, 22–24, 39). This may be due to the naturally occurring sugars in RTEC and accompanying foods such as milk and fruit, however, the contributing components cannot be clearly separated within most studies. One European study reported that RTEC breakfasts contribute to higher intakes of galactose and lactose, present in milk, but lower intake of sucrose compared to other breakfasts (18), suggesting milk may contribute to total sugar intake with RTEC breakfasts.

The contribution of RTEC to total sugar intake is also relatively modest. In the US, RTEC accounts for ≤ 4% of total sugar intake across the population (42, 43) and ≤ 12% for RTEC consumers (3, 10). Similarly, RTEC provides < 7% of total sugar intake in Australian children and adolescents (14) and < 10% of total sugar intake in Canadian RTEC consumers (9, 20).

Changes in product composition over time may also influence total sugar intake. Market assessments have shown a reduction of sugar content of cereals over time in countries such as Australia and Chile (30, 49) due to reformulation efforts and regulatory measures. In Australia, a 10% reduction in sugar content of matched cereals was observed between 2013 and 2020 (30). In Chile, the implementation of front-of-pack “high in sugar” labeling led to a sharp decrease in the number of RTEC products carrying this label, dropping from 46 to 24 within 1 year (49).

#### 3.1.5 Sodium

Observational studies generally report similar or lower sodium intake in RTEC consumers compared to non-consumers or consumers of other breakfasts (7, 8, 12–14, 20–23, 39–41). Across populations, RTEC contributes a small fraction of total sodium intake, generally 2%−3% or less (3, 10, 27) (Figure 1). For RTEC consumers, sodium intake from RTEC accounts for ≤ 8% of daily sodium intake (3, 9, 10, 14, 20).

In the US, approximately 83% of sodium in the diet comes from 20 different foods of which RTEC is not included. Reformulation efforts have further improved the sodium content of RTEC. For example, in Australia the sodium content of matched RTEC decreased by 16% between 2013 and 2020 (30).

#### 3.1.6 Saturated fat

Several observational studies have reported lower saturated fat intake in RTEC consumers compared to non-consumers or breakfast skippers (3, 7, 9, 13, 14, 36). However, some studies report no differences in saturated fat intake between RTEC consumers and non-consumers or breakfast skippers (10, 12, 20). RTEC is typically lower in saturated fat and contributes < 4% of daily intake of saturated fat (3, 7, 10, 20, 44), although when including the milk consumed with RTEC the contribution to daily saturated fat intake increases to approximately 9%−10% in the US population (3, 10).

The type of milk consumed with RTEC may influence saturated fat intake. For example, O'Neil et al. (40, 41) reported that children consuming RTEC with low fat milk had similar daily saturated fat intake as children skipping breakfast, but children consuming RTEC with full fat milk consumed approximately 2 g/d more saturated fat compared to breakfast skippers. In the US, all children exceeded the recommended amount of daily saturated fat regardless of type of breakfast intake.

### 3.2 Diet quality and food group intake

Observational studies consistently report a RTEC consumption is associated with higher diet quality, greater nutrient density and more favorable intake of whole grain, fruits, and dairy compared to non-RTEC consumers across populations (7–10, 13, 15–18, 20, 21, 40, 48, 78–82). These associations are observed in children and adults across multiple countries, including Australia, Canada, Europe, and the US. These findings may reflect the contribution of RTEC as well as an overall healthier dietary pattern among RTEC consumers.

RTEC consumers generally have higher intakes of whole grains, dairy/fluid milk, and often fruit compared to non-consumers, breakfast skippers, or those consuming other breakfasts (7, 10, 13, 18, 21, 83). Conversely, RTEC consumers also tend to have lower intakes of refined grains, but this has not been evaluated in as many countries (7, 10, 13). Evidence for intake of protein or protein foods among RTEC consumers is mixed with some studies reporting higher intakes of protein in children and adolescent RTEC consumers (18, 24), while others report no difference in children or adults (20, 38) or lower intake of protein foods (7, 10) compared to non-consumers. In Australia, RTEC consumers were also more likely to meet dietary recommendations for grains, fruits, dairy, and vegetables (13).

**Figure 3:**
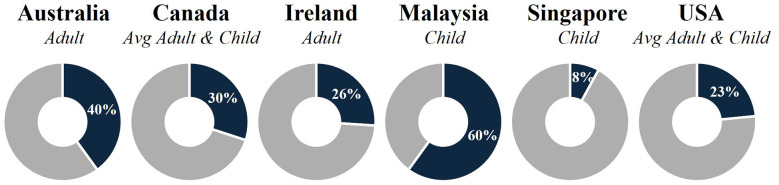
Percent contribution of RTEC to whole grains in the diets of adults and children in various countries. Data sourced from (10, 13, 20, 78, 79, 81, 82).

While higher intakes of dairy and fruit likely reflect the co-consumption of these foods, the higher intake of whole grains may also be related to the composition of the RTEC consumed. RTEC is a major contributor of whole grains in children's and adult's diets around the world, including Australia (13, 80), Canada (8, 20), Ireland (82), Malaysia (78), Singapore (81), and the US (7, 10, 17, 79) (Figure 3).

The patterns in nutrient and food group intake are reflected in higher diet quality scores and nutrient density among RTEC consumers in several populations (7, 9, 10, 13, 20, 21, 40, 83). In both the UK and France, a greater portion of adults and children with the highest diet quality include high fiber RTEC, low fat milk, and fruit in their daily diet (4, 6). In the US, diet quality was similar among children consuming RTEC breakfasts with low-fat milk, irrespective of whether the RTEC was presweetened or not (40).

Modeling studies further suggest that replacing observed breakfasts with RTEC and milk on a per calorie basis may improve overall diet quality (48). However, these findings also demonstrate that the portion size of RTEC should be considered. For example, in Ireland, the country with the highest per capita RTEC consumption (84), consumption of larger portions of RTEC (~72 g or ~2.5 servings per day) compared to smaller portions (~30 g or 1 serving per day) was associated with higher intakes of dietary fiber, iron and folate, but also higher energy density (37, 85).

### 3.3 Affordability

Observational and modeling studies suggest that RTEC may be an affordable source of nutrients and food groups. Replacing observed breakfasts with RTEC and milk in modeling studies has been associated with improvements in diet quality while also reducing the overall daily cost of the diet in the US (48). In cross-sectional analyses of children, breakfast meal costs were less for RTEC breakfasts than non-RTEC breakfast (21) and for each dollar spent, RTEC provides > 2oz equivalents of whole grain (74). RTEC consumption also improves diet quality across all income levels in Canada (20).

### 3.4 Health outcomes

While RTEC has been consistently associated with improvements in nutrient intake and diet quality, evidence linking RTEC intake to health outcomes is more heterogeneous (Table 3). RTEC intake has been investigated for its association with body weight and risk of overweight/obesity, type 2 diabetes, cardiovascular disease, cancers of the digestive tract, and all-cause mortality in multiple populations including Australia (12, 13, 61), Canada (54, 65), Chile (24), Europe (38, 57), India (35), Ireland (66), Mexico (55, 64), South Korea (26), UK (69, 72), and the US (40, 41, 47, 56, 58–60, 63, 64, 68, 86, 87). Additionally, there is emerging research on the potential benefits of RTEC for mental and emotional wellbeing and physical or mental energy (70, 71).

**Table 3:** Summary of health outcome data on RTEC.

	Body weight, BMI, and adiposity	Type 2 diabetes	Cardiovascular health and risk factors	All-cause and cause-specific mortality
Study types	Cross-sectional (*n* = 11), Cohort (*n* = 2), RCT (*n* = 4)	Cohort (*n* = 3)	Cross-sectional (*n* = 3), cohort (*n* = 3)	Cohort (*n* = 3)
Populations	Children and adults Australia, Canada, Chile, Europe, India, Ireland, South Korea, Malaysia, Mexico, USA	Adults Europe, USA	Adults Canada, South Korea, Mexico, USA	Adults UK, USA
Exposures/ Interventions	RTEC consumers vs. non-consumers; breakfast patterns; intake levels; dietary patterns; intervention vs. comparator diets	RTEC intake vs. low or no intake; whole grain RTEC; RTEC within broader dietary categories (e.g., UPF)	RTEC intake vs. low or no intake; frequency; whole grain or high-fiber RTEC; dietary patterns	RTEC intake vs. non-consumption; intake by frequency or quantity; cereal type and additions
RTEC types assessed	Total RTEC; some subtype analyses	Total RTEC; whole grain RTEC	Total RTEC; whole grain and high-fiber RTEC	Total RTEC; whole grain/high fiber; sweetened vs. unsweetened; cereal with additions
Observational findings (direction & consistency)	• Observational findings mostly report lower BMI, waist circumference, or reduced odds of overweight or obesity in RTEC consumers, but a few report no association (particularly countries with lower RTEC intake such as Malaysia and South Korea)	• Consistent but limited observational evidence • Inverse associations observed between RTEC intake and risk of type 2 diabetes, particularly for whole grain RTEC • Some evidence of mediation by fiber and minerals	• Cross-sectional studies show favorable associations of high-fiber RTEC with blood pressure and lipid profiles • Cohort studies consistently show inverse associations with CVD and CHD, primarily for whole grain or high-fiber RTEC • No associations observed for refined grain RTEC	• Inverse association of RTEC with all-cause and cardiovascular mortality, but inconsistent association with cancer mortality • Higher fiber RTEC (e.g., muesli & bran cereal) inversely associated with all-cause, CVD and cancer mortality • Sweetened cereals have neutral association with all-cause and CVD mortality and mixed associations with cancer mortality
Intervention findings (direction & consistency)	• Mixed results - weight loss observed only under energy-restricted conditions, and no consistent differences between RTEC and comparator	• No interventional evidence identified	• No interventional evidence identified	• No interventional evidence identified

#### 3.4.1 Body weight

Observational research in the past decade examining the relationship between RTEC intake and body weight continues to report mixed findings with several studies showing inverse associations with body weight, BMI, or risk of overweight/obesity (12, 13, 24, 38, 40, 41, 54, 55), while others report no association (9, 14, 19, 26, 54, 67). However, no studies have reported a positive association of RTEC with body weight or obesity. Additionally, in children, the associations of RTEC with body weight are similar with presweetened and non-presweetened cereal (40, 86). While most studies attempt to adjust for various confounders, such as age, sex, energy intake and physical activity, residual confounding cannot be excluded, and these findings may reflect broader dietary and lifestyle factors among RTEC consumers.

Intervention studies may help provide additional context but are limited in number and vary in design (35, 47, 61, 66). Three studies using RTEC as a meal replacement reported a reduction in overall energy consumption and body weight (35, 61, 66). When compared to a normal diet or dietary advice for weight loss, studies providing RTEC as a meal replacement for lunch and dinner for 2 weeks demonstrate significantly more weight loss (35, 66). However, when RTEC was compared to an alternative breakfast (e.g., eggs) with similar caloric restriction (61), the weight loss was similar between the two diets, suggesting that energy restriction is likely the driver of weight loss rather than breakfast composition. These findings suggest RTEC can be useful as a meal replacement within a weight loss diet but likely does not have an effect independent of energy reduction.

An additional study in adolescent girls consuming one serving of fortified or unfortified RTEC in the morning or the evening reported no body weight differences between the groups after 12 weeks of consumption (47). However, the group consuming RTEC in the evening gained a small but significant amount of weight during the 12-week intervention, while those consuming RTEC in the morning did not, warranting further investigation into the implications of meal timing on body weight.

#### 3.4.2 Type 2 diabetes & glycemic control

Recent prospective cohort studies in the US and Europe report inverse associations between RTEC intake and risk of type 2 diabetes in adults (56–58). These studies differ in the RTEC exposure investigated with one study examining whole grain RTEC specifically (58), while the other examined total RTEC intake as a subset of ultra-processed foods. Hu et al. (58) examined whole grain RTEC intake and reported that individuals consuming one or more servings of whole grain RTEC (≥ 25% whole grain or bran by weight) per day had a lower risk of type 2 diabetes than those consuming less than a serving a month. The other two studies found similar associations when examining total RTEC intake but did not differentiate whole grain and refined grain cereals. However, Chen et al. (56) reported that fiber and mineral content of RTEC mediated some of the observed relationship between RTEC intake and type 2 diabetes risk.

Taken together, this suggests that the nutrient content (fiber and minerals) and food groups (whole grain) provided by RTEC may partially contribute to the association of RTEC and reduced risk of type 2 diabetes. However, there may also be other dietary and lifestyle confounders that cannot be completely adjusted even though all studies included a broad list of covariates.

#### 3.4.3 Cardiovascular health

Prospective cohorts and cross-sectional studies report inverse associations of RTEC consumption with risk of coronary heart disease, cardiovascular disease, stroke, and hypertension (26, 59, 60, 63–65), although a few of these studies only examined whole grain or high-fiber RTEC. In two prospective cohorts, intake of RTEC, was associated with a lower risk of cardiovascular disease, coronary heart disease, and stroke but stratification by type of RTEC was not done (63, 65). Two additional cohort analysis reported consumption of whole grain (or bran) RTEC was associated with a lower risk of coronary heart disease (1 svg/month to >1svg/d) and ischemic stroke (>1 svg/wk) compared to those consuming less than one serving per month, but neither analysis examined the association with refined grain RTEC (59, 60). Evidence for cardiovascular risk factors show a similar pattern to disease endpoints (26, 64). RTEC consumption was inversely associated with blood pressure, and the prevalence of hypertension was reported as significantly lower in RTEC consumers compared to non-consumers (26). However, there was no examination by type of RTEC. Consuming more than one serving per week of high-fiber breakfast cereal was associated with a decreased risk of hypertension when compared to ≤ 3 times per month or never, whereas refined grain RTEC intake was not associated with an increased risk of high blood pressure (64).

Across these studies, RTEC intake, particularly higher fiber or whole grain RTEC was associated with lower risk of cardiovascular outcomes and risk factors. While the studies adjusted for many covariates such as age, sex, energy intake, BMI, physical activity, and healthy eating index, there remains the possibility of residual confounding.

#### 3.4.4 Mortality

Three prospective cohort studies in the US and UK have evaluated the relationship of RTEC intake and mortality from all-causes and from specific causes, such as CVD, cancer, and diabetes (68, 69, 72). These studies report RTEC consumption is associated with reduced risk of all-cause mortality, but the findings differ based on the type of RTEC, particularly the fiber and sugar content.

A large cohort of more than 360,000 individuals in the US followed for an average of 14 years, reported that participants in the highest quartile of RTEC consumption (~22 g/d) had lower risk of all-cause mortality, CVD mortality, all-cancer mortality, and digestive cancer mortality compared to non-consumers of RTEC (68). Among RTEC consumers, those with the highest fiber intake (~26 g/d) had reduced risk of all-cause mortality, CVD mortality, all-cancer mortality, and digestive cancer mortality compared to the lowest fiber consumers (~9 g/d), indicating that fiber content may contribute to the observed associations. Similarly, in a UK cohort, RTEC consumption was associated with lower risk of all-cause, and CVD mortality, but not cancer mortality, compared to non-consumers of RTEC (72). When types of RTEC were examined, moderate consumption of muesli or bran RTEC (≤1 bowl/d), was associated with lower risk of all-cause, CVD, and cancer mortality compared to non-consumers of these types of RTEC, although these associations were attenuated at higher intake levels (>1 bowl/d).

Addition of sweeteners may also influence observed associations. Sweetened RTEC consumption was not associated with increased risk of CVD or all-cause mortality, but consumption of more than half a bowl per day was positively associated with risk of cancer mortality (72). Zhang et al. (69) compared consumers who added sweeteners to their cereal vs. those who do not (in the study it is called “unsweetened cereal” but may include sugars added by the manufacturer). Individuals who did not add sweeteners to their cereal had a lower risk of all-cause mortality and CVD mortality which was not observed in individuals adding caloric or non-caloric sweeteners to their cereal.

While there is a limited number of studies, prospective cohort evidence suggests RTEC intake is associated with lower risk of all-cause and CVD mortality, but these associations vary based on RTEC type and patterns of consumption. Several covariates were adjusted for in these studies, but residual confounding due to dietary or lifestyle patterns cannot be ruled out.

#### 3.4.5 Other health outcomes

Emerging research has evaluated the role of RTEC in emotional wellbeing and physical/mental energy. While most research in this area compares breakfast consumption to breakfast skipping, one study evaluated emotional wellbeing in children and adults provided a low-fiber or high-fiber RTEC for 14 days (70). Compared to breakfast skipping, individuals consuming RTEC breakfasts reported less depression, anxiety, emotional distress, fatigue, and cognitive difficulties; however, there were no differences between the types of RTEC except for greater alertness with a high-fiber RTEC.

Similarly, another study reported improvements in fatigue over 2–4 weeks with an RTEC breakfast compared to skipping breakfast but did not include a comparison between types of RTEC or different types of breakfast meals (71). A separate study reported improvements in cognitive parameters in 11–13 year old children consuming an RTEC breakfast, but the comparator was no breakfast, thus the benefits observed may be due to consumption of breakfast rather than specific effects of RTEC (73).

## 4 Discussion

Over the past decade, research continues to support associations between RTEC consumption and higher nutrient intake, better diet quality, and certain positive health outcomes around the world. Research across diverse populations suggests that RTEC may provide an affordable, and accessible source of nutrition, contributing 10%−20% of several key nutrients and dietary fiber for children and adults in multiple countries. RTEC consumption is also associated with higher intake of key food groups, including whole grains, dairy, and fruit, which contributes to better diet quality and provides a balance of nutrients and food groups for breakfast. Furthermore, RTEC, particularly whole grain or high-fiber RTEC, is frequently associated with favorable chronic health outcomes, including reduced risk of cardiometabolic diseases. RTEC contributes relatively little to saturated fat and sodium intake, but total sugar intake is higher among RTEC consumers. This may be partially explained by inherent sugars in RTEC, milk and/or fruit as added sugar intake is similar to other breakfasts or breakfast skippers. Importantly, much of the available evidence for RTEC consumption is observational, with a limited number of intervention studies. Therefore, the observed associations may also reflect broader dietary and lifestyle patterns among RTEC consumers, although most studies adjusted for several covariates, such as age, sex, BMI, energy intake, and physical activity in their analyses. Conclusions about causality should be made with caution.

Consistent with previous research reviews, RTEC consumers around the globe are more likely to meet micronutrient requirements and have higher dietary fiber intake than non-consumers indicating the role of RTEC and RTEC fortification as key contributors to nutrient adequacy and sources of shortfall nutrients across populations (3, 7, 9–14, 16–25, 29, 31, 32, 38, 39). Intervention studies with fortified RTEC also demonstrate an improvement in micronutrient status, particularly vitamin B12, vitamin D, folate, and iron (47), but more studies are needed, particularly in countries with higher prevalence of micronutrient inadequacy. Modeling studies suggest that removal of fortification from RTEC may increase the prevalence of nutrient inadequacies across multiple micronutrients (31), while replacement of non-RTEC breakfast with fortified RTEC could increase iron, folate, and dietary fiber intake (48). There have been concerns about potential over-consumption of folate due to RTEC fortification, but studies indicate that typical intake levels with RTEC consumption do not pose a significant risk of exceeding the upper limit for folate (33, 51). Additionally, while most individuals around the world do not meet fiber recommendations, RTEC can help mitigate this gap and support overall dietary quality.

While the saturated fat content of RTEC has historically been lower than most other breakfast foods, the sodium and added sugar content of RTEC has received more attention from consumers and regulators, leading to reformulation to reduce these nutrients. In several countries there has been progress in reducing sodium and added sugar; however, there is still room for continued improvement, particularly for added sugar (88–90). The population level data presented here suggests RTEC is not a major contributor to sodium, added sugars or saturated fat. At the individual level, however, the contribution of RTEC to added sugar will vary depending on the type of RTEC consumed as added sugar levels can vary considerably across products. Total sugar intake is generally higher in RTEC consumers, in agreement with previous reports (1, 2), although this may be due to both the sugar content of RTEC as well as inherent sugars in foods co-consumed with RTEC, such as milk and fruit.

Interest in level of food processing and ultra-processed food has increased in recent years. RTEC has been classified as ultra-processed or highly processed foods within certain food categorization frameworks (56, 63, 91). This may raise questions regarding their role in healthy dietary patterns. Studies examining the association of different types of ultra-processed foods reported inverse associations of RTEC with health outcomes, such as CVD risk and T2D (57, 63). The findings of this review suggest that across global populations, RTEC consumption is associated with higher nutrient intake, diet quality, and favorable health outcomes, particularly for RTEC higher in whole grain and dietary fiber. At the same time, the nutrient content of RTEC can vary considerably, demonstrating the importance of distinguishing between different types of RTEC in observational and intervention studies rather than treating them as a homogenous category.

While there has been considerable new research emerging around the globe on the nutritional benefits of RTEC, there remains a gap in data from many countries. A lack of large national data sets in most countries means most data must be obtained from much smaller and local samples and may not reflect the intake of the larger population. Also, nutrient databases are frequently lacking data on many local or regional food items as well as data on many micronutrients. To understand the role of RTEC more accurately on nutrient intake in populations around the world, there is a great need for national data sets and food analyses. These can be costly to initiate and maintain but provide critical information that can impact public health.

As there has been a transition to food based dietary guidelines in the past decade, there has also been an increase in research on the role of RTEC in improving diet quality and increasing food group consumption. Studies consistently report that RTEC consumption is associated with higher diet quality and contributes to whole grain, dairy and fruit intake across the globe (7–10, 13, 15–18, 20, 21, 40, 48, 78–83). Some regions have recommendations for a “good,” “balanced,” or “quality” breakfast which includes a grain food, dairy food, and fruit (4, 83, 92, 93). RTEC with milk and a piece of fruit aligns with these recommendations and provides a variety of food groups and nutrients. Guidance also suggests opting for whole grain or high fiber grain foods and minimizing foods with added sugars.

Additionally, there has been an increase in research on the affordability of RTEC and its association with improved nutrient intakes across all income groups, which was not included in previous reports. Given that many of the health outcomes evaluated in this review disparately impact individuals with lower incomes who may experience food insecurity and thus poor diet quality (20, 21), access to affordable, nutrient-dense foods is a potential strategy to support diet quality. RTEC consumption was associated with higher diet quality, even in food insecure or low-income households (20, 94). However, additional research on the affordability of RTEC in low and middle income countries is needed as current research is limited to high-income countries.

Similar to previous literature (1, 2, 86, 87), RTEC intake was associated with reduced prevalence and risk of overweight or obesity and healthier markers of body weight and adiposity, including BMI and waist circumference in some studies, but the findings were not consistent. Several mechanisms have been proposed to explain the observed associations between RTEC consumption and healthier body weight, but to date, no clinical trials have conclusively established a cause-effect relationship or mechanism by which RTEC may influence body weight (86, 87). A reduction in energy intake is the most common explanation for RTEC's association with body weight, but most observational studies report mixed results on the association of RTEC with energy intake and results from clinical trials on appetite and energy intake have been inconclusive (35, 62). RTEC consumption has been associated with healthy lifestyle habits, such as a healthier diet and more physical activity which may also contribute to healthier weight status. The dietary fiber content of RTEC may also play a role as one longitudinal study reported a reduced risk of obesity in consumers of high fiber cereals, including All Bran^®^, muesli, and oat-based cereals, which was not observed with overall RTEC consumption (19). Alternatively, the timing of RTEC consumption may influence body weight and this deserves more investigation. In the intervention study by Powers et al. (47), adolescent girls who consumed RTEC in the evening gained a small but significant amount of weight during the 12-week intervention, while those consuming RTEC in the morning did not. This is consistent with data demonstrating greater weight loss with higher energy intake at the morning meal compared to the evening meal (95).

The reviewed studies also demonstrated a reduction in risk of type 2 diabetes, CVD, CHD, stroke, and a reduction in mortality from CVD, cancer, type 2 diabetes, and all causes; however, some of the associations varied by RTEC composition. Associations with reduced risk were more consistent with high fiber or whole grain RTEC. Chen et al. (56) estimated that up to 18.5% of the inverse relationship of RTEC and incident type 2 diabetes could be attributed to the fiber or mineral content of the RTEC. Two of these studies also examined RTEC as a distinct food category within the broader categorization of ultra-processed foods and RTEC was inversely associated with risk of type 2 diabetes and cardiovascular disease, in contrast to other ultra-processed food subcategories (57, 63). Notably, the number of recent prospective studies examining RTEC and chronic disease risk remains limited with a relatively small number identified since 2015, but the data are consistent with broader evidence linking whole grains and dietary fiber to reduced risk of type 2 diabetes and CVD.

The purpose of this review was to provide an update to previous reviews conducted in 2015 and 2016 (1, 2) including research in other parts of the world where RTEC is being adopted for breakfast. While the inclusion and exclusion criteria were slightly different between the previous reviews and the present review, the results remain broadly consistent, demonstrating associations between RTEC consumption and higher vitamin, mineral, and dietary fiber intake, better diet quality, and favorable health outcomes. However, most research continues to focus on nutrient intake with health outcome studies generally conducted in Europe, the UK, and US. Thus, there is a need to expand health outcome research to other countries, particularly low- or middle-income countries where there may be greater nutrient insufficiencies and a growing prevalence of chronic disease.

Additionally, both previous reviews reported that the benefits of RTEC for outcomes such as cognition, mental/physical energy and overall wellbeing were emerging, and more research was needed. However, there has been little progress in this area over the past 10 years. The current review found no new studies on RTEC and cognition and only two studies on mental/physical energy and wellbeing (70, 71). Both studies report differences between RTEC consumption and skipping breakfast but only one examined differences between types of RTEC and neither compared to alternative breakfasts. It is possible that the benefits observed are due to consuming breakfast rather than the type of breakfast, but this cannot be concluded without additional research.

This review has several strengths including a focus on the past 10 years of research to evaluate more current evidence for differences and alignment with older research. Additionally, broad search terms with no search restrictions for outcomes were used to ensure a comprehensive scope of the literature, including research on affordability and mental/emotional wellbeing. However, there were also limitations to this review, such as not employing systematic methodology or risk of bias assessments which would add to the strength of the analysis. Additionally, much of the research reviewed is observational in nature which is subject to confounding and reverse causality. For this reason, more clinical trials are needed to establish the causality of RTEC in relation to health benefits. Future observational and clinical trials should also include better stratification by RTEC composition as several of the studies in the current review, particularly health outcome studies, report differing associations based on nutritional composition.

## 5 Conclusion

The past decade of research has continued to support associations between RTEC consumption and higher nutrient intake and better diet quality around the world. As a nutrient-dense, affordable, and accessible food, RTEC may contribute to addressing global nutrient needs and supporting overall diet quality. Despite concerns about sugar and sodium content, the recent evidence indicates RTEC is a small contributor of both in the diet. When consumed as a balanced breakfast with milk and fruit, RTEC consumption may provide a practical way to increase intake of key nutrients and food groups and support overall diet quality across populations.
