# Supplementary material for: The nutritional and health benefits of ready-to-eat-cereal consumption: an updated technical review of global evidence
Source: Front Nutr. 2026 Jun 17;13:1778338. doi: 10.3389/fnut.2026.1778338 (PMC13318615; doi:10.3389/fnut.2026.1778338)
Supplement: Supplementary file 1 [file Table_1.docx]

Supplementary Material

| **Reference** | **Design** | **Location** | **Population/ Age** | **Exposure/**  **Intervention** | **Comparator** | **Outcomes** | **Covariates assessed** |
| --- | --- | --- | --- | --- | --- | --- | --- |
| **Nutrient Intake** | | | | | | | |
| Afeiche, 2017 | Cross-sectional | Mexico | Children  4 – 13 y | RTEC breakfast | Other breakfasts and skipping breakfast | - Across all breakfast dietary patterns, children in the cereal and milk breakfast dietary pattern had the highest intakes of B vitamins (except folate), vitamin D, zinc, and iron at breakfast and for the total day. - In addition to those listed above, RTEC consumers also had higher intake of calcium compared to skippers. - RTEC consumers had higher total sugar and added sugar intakes compared to skippers. - RTEC consumers had similar sodium intake to skippers and other breakfasts. | child age, sex, weight status, residence (rural vs. urban), region, and socioeconomic status (SES) tertiles |
| Calame, 2020 | Modeling | UK | All ages  4 – 65+y | Fortification of Vitamin D | No fortification | - Fortification of RTEC with vitamin D (4.2 ug/ 100 g RTEC) could increase vit D status from 1.0 nmol/L (children) to 6.5 nmol/L in elderly | age, gender, BMI, time of the year, daily intake of vitamin D, daily intake of RTEC, daily intake of other dietary factors, and economic status |
| Castillo Valenzuela, 2015 | Cross-sectional | Chile | Children  6 – 13 y | RTEC consumption  0 g/d (non-consumers), 1-29 g/d (1 portion or less), 30-59 g/d (1 to 2 portions), and ≥60 g/d (2 or more portions) | Lower or no RTEC consumption | - RTEC consumption was positively associated with intake of all macronutrients and insoluble fiber, vitamin A, B6, C, D, calcium, iron and zinc. - RTEC consumption positively is associated with intake of total sugars, energy, and sodium | gender, age, pubertal maturation, physical inactivity (hours per day in front of a screen), and socioeconomic level |
| Chung, 2015 | Cross-sectional | Korea | Children and adults  1 – 75 y | RTEC consumption | Non-RTEC consumption | - RTEC consumers had significantly higher intakes of calcium, thiamin, riboflavin and vitamin C | age, gender, education, residential area, and occupation |
| Cuadrado-Soto, 2018 | Cross-sectional | Spain | Children  7 – 11 y | Sodium in the diet | NA | - Breakfast cereals provided 2.2% of sodium in the diet of children. | none |
| Fayet-Moore, 2018 | Cross-sectional | Australia | Children and adults  2 – 18 y  ≥ 19 y | Fiber intake | NA | - RTEC was in top 3 sources of dietary fiber in high fiber consumers, but not low fiber consumers - Low-sugar wheat based breakfast cereal was leading fiber contributor in high fiber consumers (adult only) - Higher intake of fiber associated with high intake of WG, F&V | energy intake |
| Fayet-Moore, 2016 | Cross-sectional | Australia | Children  2 – 16 y | RTEC for breakfast | Skipping  Non-cereal breakfast | RTEC consumers more likely to meet nutrient requirements and consume more fiber, Ca, Fe, folate, total sugars, and CHO, and lower fat and Na compared to non-cereal | age, sex, and physical activity level |
| Fayet-Moore, 2019 | Cross-sectional | Australia | Adults  ≥ 19 y | RTEC for breakfast | Skipping  Non-cereal breakfast | - RTEC consumption increased with age - RTEC consumers had healthier diets compared to non-RTEC and skipper (lower added sugar, lower total and sat fat; higher CHO & fiber and all micronutrients except Na) (fiber due to higher intake at bf) - Skippers had highest intake of added & free sugars - Relative to its energy contribution, breakfast cereal contributed half the total fat, almost one and a half times as much carbohydrate, half the sodium, at least double the fiber, riboflavin, and folate, and more than triple the iron and thiamin | sex, age group, their interaction, BMI group, and energy intake at total day, breakfast, or the rest of the day |
| Fayet-Moore, 2017 | Cross-sectional | Australia | Children & adolescents  2 – 18 y | RTEC breakfast consumers  minimally pre-sweetened(MPS) < 15% or pre-sweetened (PS) >15% or PS>30% | Non-cereal breakfast consumers  skippers | - RTEC consumers had lower sat fat intake and higher fiber and micronutrient intake compared to skippers - RTEC consumers had similar added sugar intake, lower Na, and higher total sugars, CHO, fiber and most other micronutrients compared to non-cereal breakfast consumers - Only nutrient difference for presweetened and minimally presweetened was higher folate intake in presweetened consumers - total and added sugars intake not different between MPS and PS (>15% and >30%) even though PS contributed greater portion total/added sugars to the overall diet - MPS consumers added more sugar to the cereal bowl (5.2g)than PS consumers (0g). | age, sex, BMI z-score, and energy intake |
| Fulgoni, 2019 | Cross-sectional | USA | Children & adolescents  2 – 18 y | Low sugar ≤ 21.2% and high sugar >21.2% RTEC breakfast | Oatmeal | - RTEC consumers have higher intakes of iron and folate compared to oatmeal, but lower intake of whole grain, magnesium, and fiber (magnesium and fiber only different from high sugar RTEC) | age, gender, race/ethnicity, poverty income ratio, physical activity level, and kcal |
| Fulgoni, 2015 | Cross-sectional and modeling | USA | Children and adults  2 – 18 y  19 -99 y  2 – 99 y | Fortified RTEC | Non-fortified RTEC (not actual intake but subtracting contribution of RTEC to V&M intake) | - Without fortification, the portion of children in the population below EAR or AI significantly increases for folate, iron, niacin, vitamin B2, thiamin, vitamin A, vitamin B6, vitamin D, vitamin E and zinc - Without fortification, the portion of adults in the population below EAR or AI significantly increases for calcium, folate, iron, magnesium, niacin, riboflavin, thiamin, vitamin A, B12, B6, E and zinc. - Without fortification the portion of the total population below EAR or AI significantly increases for calcium, folate, iron, magnesium, niacin, riboflavin, thiamin, vitamin A, B12, B6, C, D, E and zinc | in the NCI usual intake estimation: sequence coded by day (weekend or weekday) and DRI age groups (2–18, 19–99 and 2–99 years) |
| Hennessy, 2017 | Cross-sectional | Ireland | Preschool children  1 – 4 y | Fortified foods (including RTEC) | Non-fortified foods and supplements | - Children who did not consume fortified foods, including RTEC had lower intake of vitamin D - RTEC contributed ~0.3 ug vitamin D/d to those who consumed it (24% of children consumed RTEC) - Even with fortified foods and supplements, the majority of children still don’t meet vitamin D needs - There is little risk of excessive intake of vitamin D from fortified foods | none |
| Kehoe, 2023 | Cross-sectional | Ireland | School children  5 – 12 y | RTEC intake (high fiber and low fiber) | NA | - From 2003 – 2004 to 2017 – 2018, intake of high fiber cereals increased, and intake of low fiber cereals decreased | sex and age group (5-8 years and 9-12 years) |
| Koo, 2015 | Cross-sectional | Malaysia | School children  10 – 11 y | RTEC intake | Skipping breakfast  Non-cereal breakfast | - 20.5% of children reported consuming RTEC for breakfast during the 24 hr recall and 93% reported the consumed RTEC sometime during the week - RTEC consumers had higher intake of calories, CHO, vitamin A, B1, B2, B3, C, folate, calcium, iron and fiber compared to skippers and those who had other breakfasts | none |
| Kuriyan, 2017 | RCT | India | Adult women with ow/ob  18 – 44 y | Low-fat RTEC breakfast and lunch for 2 wk | Dietary advice for wt loss | - Intakes of niacin, vitamin B-6, B-12, fiber and sugar were significantly greater in the intervention group | NA |
| Lepicard, 2017 | Cross-sectional | France | Children  9 – 11 y | RTEC + milk breakfast pattern | Other breakfast patterns (sweets, traditional French bread & butter, dairy + juice) | - Nutritionally, the RTEC + milk pattern was the most advantageous. The mean adequacy ratio for nutrients was highest for the RTEC + milk pattern - RTEC breakfast had the lowest energy density compared to other breakfasts - RTEC + milk breakfasts had the lowest fat, sat fat, cholesterol and highest calcium, iron, potassium, vitamin A, C, thiamin, riboflavin (B2), niacin (B3), pantothenic acid (B5), B6, folate, and B12 of all breakfast patterns | Sex assessed separately, total energy intake |
| Lyons, 2018 | Cross-sectional | Ireland | Adults  18 – 64 y | Portion sizes of RTEC and energy density  Tertiles of portion sizes were 30 g, 45 g, and 72 g | NA | - Energy density was higher on days when larger portions of RTEC were consumed - Iron, folate, B vitamins and fiber intake was higher on days when larger portions of RTEC were consumed | energy intake |
| McGill, 2016 | Cross-sectional | USA | Adults  >19 y | Meeting the AI for fiber | Not meeting AI for fiber | - Grain products were a major contributor to fiber intake and RTEC was one of the top grain products. - RTEC contributed 22.5% of the fiber in individuals meeting the AI for fiber | age, gender, and ethnicity |
| McGill, 2015 | Cross-sectional | USA | Adults and children  4 – 18 y  19 – 50 y  ≥ 51 y | Food sources of WG and fiber | NA | - RTEC was a major contributor of WG (31% of WG was from RTEC) in children/ adolescents and adults (23% of WG from RTEC) - No improvements in WG or fiber intake from RTEC from 2001 - 2010 | age, gender, race-ethnicity, socioeconomic status, and education |
| Michels, 2016 | Cross-sectional | Europe | Adolescents  12.5 – 17.5 y | RTEC breakfast | Bread breakfast  Other non-RTEC breakfast | - RTEC breakfast associated with lower fat and sucrose intake and higher fiber, protein, vitamin B, calcium, magnesium, potassium, phosphorous, and vitamin C compared to bread breakfast, but lower Na, vitamins A, E and K - RTEC associated with lower sat fat and lower sugar compared to other breakfast and higher protein, fiber, B vitamins, calcium, magnesium, phosphorous, vitamin C and K, iron, Na - RTEC breakfast had lower energy content than bread breakfast but higher energy content than other breakfasts - RTEC breakfast associated with higher glucose and fructose intake compared to bread breakfast - RTEC consumers had higher frequency of fruit and dairy intake with breakfast - Regarding simple sugars, an RTEC breakfast provided higher relative intake of galactose and lactose, but lower intake of sucrose than “all other breakfasts” and “bread breakfast”. | gender, age, socio-economic status, and city |
| Michels, 2015 | Cross-sectional | Europe | Adolescents  12.5 – 17.5 y | RTEC consumption frequency  (once/wk, 2–4 times/wk, ≥ 5-6 times/wk) | No-RTEC consumption  (never or < once/wk) | - RTEC consumers had higher intakes of vitamin B2, B5, B7, D, calcium, phosphorus and potassium (linear regression across frequency) - No differences were noted in macronutrients or energy intake | sex, age, socio-economic status, city, and breakfast skipping |
| Mohd Nasir, 2017 | Cross-sectional | Malaysia | Children  6 – 12 y | RTEC consumption | No-RTEC consumption | - RTEC contributed 10% of daily calcium and 15% of daily iron and ≥ 20% daily vitamin C, thiamin, riboflavin, niacin (total pop) - RTEC consumers had lower intakes of fat and Na than no RTEC consumers at breakfast and throughout the day - Total sugar consumption was higher with RTEC consumers | socio-economic status and energy intake |
| O’Neil, 2015 | Cross-sectional | USA | Children  2 – 18 y | RTEC consumption patterns (including presweetened RTEC and whole milk, presweetened RTEC and low fat milk, RTEC and whole milk, RTEC and low fat milk) | Skipping breakfast | - All RTEC patterns had higher energy consumption than skippers - Added sugars intake did not differ from breakfast skippers for any RTEC patterns, except RTEC + whole milk which had lower added sugar intake than skippers - Sat fat intake was lower than skippers for presweetened RTEC + low fat milk but higher for RTEC and whole milk - No differences in Na intake - Presweetened and non-presweetened RTEC with low fat milk had higher fiber intake compared to skippers - All RTEC groups had higher intakes of vitamin D, Ca, potassium, vitamin A, folate, iron and magnesium - Presweetened and non-presweetened RTEC with whole milk had lower BMI z-score compared to skippers (no diff with RTEC + low fat milk) | self-reported age, gender, race/ethnicity, and poverty income ratio (PIR) grouped into three categories (< 1.25, 1.25–3.49, and > 3.49), physical activity, energy intake |
| Papanikolaou, 2017 | Cross-sectional | USA | Adults  ≥ 19 y | Grain food consumption | NA | RTEC nutrient contribution for Adult females (some from graph)  Folate (DFE): 11.3%, Iron: 10.6%, Vitamin B12: 9.0%, Vitamin B6: 8.8%, Thiamin: 7.7%, Niacin: 7.2%, Vitamin A: 6.9%, Riboflavin: 6.1%, Zinc: 6.1%,  Dietary Fiber: 5.0%, Vitamin D: 4.9%,  Carbohydrates: ~3.8%, Magnesium: ~3.2%, Vitamin E: ~2.9%, Total Sugar: 2.6%, Energy (kcal): 2.4%, Phosphorus: ~2.3%, Sodium: 1.8%, Calcium: ~1.8%, Potassium: ~1.6%, Polyunsaturated Fat: ~1.5%, Protein: ~1.4%, Monounsaturated Fat: ~1.2%, Total Fat: 0.9%, Saturated Fat: 0.6%  RTEC nutrient contribution for Adult males (some data in text does not match graph, graph data used)  Folate (DFE): ~11.0%. Iron: ~10.0%, Vitamin B6: ~8.4%, Vitamin B12: ~8.3%, Thiamin: ~7.3%, Vitamin A: ~6.9%, Niacin: ~6.5%, Riboflavin: ~6.0%, Zinc: ~5.9%, Vitamin D: ~5.0%, Dietary Fiber: ~5.1%, Carbohydrates: ~3.7%, Vitamin E: ~3.0%, Magnesium: ~2.8%, Total Sugar: 2.7%, Phosphorus: ~2.6%, Energy (kcal): 2.1%, Sodium: 1.7%, Calcium: ~1.7%, Potassium: ~1.6%, Polyunsaturated Fat: ~1.4%, Protein: ~1.3%, Total Fat: 0.8%, Monounsaturated Fat: ~0.9%, Saturated Fat: 0.5% | survey weights to generate nationally-representative estimates |
| Papanikolaou, 2017 | Cross-sectional | USA | Children & Adolescents  2 – 18 y | Grain food consumption | NA | RTEC nutrient contribution in children and adolescents (some data from graph)  Folate (DFE): 18.2%, Iron: 16.6%, vitamin B6: ~14.8%, Vitamin B12: 13.0%, Niacin: 12.5%, Thiamin: 12.3%, Vitamin A: 11.7%, Zinc: 10.3%, Riboflavin: ~10.1%, Vitamin D: 7.3%, Dietary Fiber: 6.5%, Carbohydrates: ~5.0%, Total Sugar: 4.0%, Magnesium: ~3.8%, Energy (kcal): 3.3%, Vitamin E: ~3.2%, Phosphorus: ~3.0%, Sodium: 3.0%, Calcium: ~2.6%, Polyunsaturated Fat: ~2.3%, Potassium: ~1.9%, Protein: ~1.8%, Monounsaturated Fat: ~1.6%, Total Fat: 1.2%, Saturated Fat: 0.9% | survey weights to generate nationally-representative estimates |
| Papanikolaou, 2018 | Cross-sectional | USA | Older adults  ≥ 51 y | Grain food consumption | NA | - RTEC contributes 2.35% of daily energy intake - RTEC contributes 21% of daily folate intake (top source) - RTEC contributes 1/3 of daily iron intake (top source) - RTEC was a top 10 contributor to magnesium, fiber, vitamin A, thiamin, vitamin B6 and vitamin B12, niacin - RTEC contributes < 2.5% of Na, < 1% total fat, < 0.5% sat fat, < 4% added sugar | survey weights to generate nationally-representative estimates |
| Papanikolaou, 2021 | Modeling | USA | Adults  19 – 50 y  51 – 99 y | Nutrient intake with grain foods included in diet | Nutrient intake without grain foods | - Removal of 25%, 50%, and 100% of grains resulted in only 2.6%, 1.8%, and 0.7% of adults exceeding the AI for fiber - If all grains were removed, 43.4% and 56.2% of younger and older adults would be below the EAR for folate (currently 11% and 13.8%) - If all grains were removed, 10% and 22% of younger and older adults would be below the EAR for iron (currently 8.4% and 0.8%) - If all grains were removed, 68% and 73% of younger and older adults would be below the EAR for magnesium (currently 51% and 54%) | Age groups, gender, day sequence, weekend |
| Park, 2023 | Cross-sectional | USA | Children and adolescents age 2 – 19 y | Sources of added sugars in high added sugar consumers (>15% kcal from added sugar) | NA | - In youth consuming >15% of calories as added sugar (approx. 1/3 of sample), RTEC contributed ~5% of daily added sugar calories (22 kcal/d). | age, sex, race/ethnicity, head of household education, federal poverty income ratio, and weight status |
| Powers, 2016 | RCT | UK | Adolescent girls  16 – 19 y | 50 g fortified cereal + 150 mL skim milk for 12 wk (breakfast or evening snack) | 50 g unfortified cereal + 150 mL skim milk for 12 wk (breakfast or evening snack) | - Unfortified cereal consumers increased intake of B1, B2, and B6 - Fortified cereal consumers increased intake of B1, B2, B6, B12, folate, iron and vitamin C and D compared to unfortified cereal consumers - Biomarkers of status for B2, B12, folate, and iron were improved in fortified vs unfortified RTEC consumers - Vitamin D status was maintained in fortified vs unfortified RTEC consumers | NA |
| Quatela, 2017 | Prospective cohort (12 y follow up) | Australia | Middle-aged women  Mean 52.4 y  45 – 50 y at initiation of cohort | RTEC consumers  Also specific categories (All-Bran, Sultana Bran/Fibre Plus/Branflakes, Weet Bix/Vita Brits/Weeties, Cornflakes/Nutrigrain/Special K, muesli, oat-based, wheat based, higher fiber or whole grain | RTEC non-consumers | - RTEC consumption associated with higher fiber intake (20.4 vs 16.5 g/d) – highest in All-Bran group - RTEC consumption associated with higher energy intake (~600 kcals/d) | area of residency, income, smoking, physical activity, hypertension, daily energy intake, daily fiber intake, and other breakfast cereal consumption |
| Rehm, 2017 | Modeling | USA | Adults & children  ≥ 1 y | Replacement of breakfast foods with RTEC on per calorie basis  (RTEC alone and RTEC + milk) | Observed breakfast | - Replacement with RTEC alone increased WG, fiber, vit D, iron and folate. Also increased added sugar and decreased sat fat - Replacement with RTEC + milk increased WG, fiber, vit D, iron, folate, calcium, potassium and dairy food group. Also increased added sugar and decreased sat fat - % energy from added sugars and sat fats decreased in both models - Diet quality higher with replacement with RTEC + milk | recall data (weekday/weekend), recall order (first/second), and mode (telephone/in-person) |
| Sanders, 2023 | Cross sectional | Canada | Adults and children  2 – 18 y  19+ y | RTEC consumption by income level | No RTEC consumption | - RTEC consumers had higher intakes of iron, calcium, fiber, magnesium, vit D - RTEC consumers had higher total sugar intake and lower fat and chol intake; no difference in sat fat, protein, or Na - RTEC provided < 10% of energy intake < 4% sat fat intake and <9% total sugar intake, 1/3 daily iron intake and 10% of fiber, thiamin, folate, and B6 (total population). - No differences in daily energy intake based on RTEC consumption | age, sex, and energy intake |
| Smith, 2022 | Cross sectional | USA | Adults and children  2 – 18 y  ≥ 19 y | RTEC consumption by income level | No RTEC consumption | - RTEC consumers had a higher intake of carbohydrates, fiber, total sugar, calcium, iron, magnesium, phosphorus, potassium, zinc, folate, niacin, riboflavin, thiamine, vitamins A, B6, B12, C (adults only), and D, total dairy, fluid milk, total fruit (adults only), intact fruit (adults only) and WG. - RTEC consumers had lower intake of total fat, saturated fat, selenium, sodium, refined grains (children only), total protein foods, and total meat, poultry, seafood, and egg intake - RTEC consumption was significantly associated with higher diet quality for children and adults. - RTEC contributed ∼10% to daily energy intake across all ages. RTEC contributed to one third or more of daily intake, across all ages for folate, iron, WG, and vitamins B6 and B12 | age, energy intake, gender, and race/ethnicity |
| Smith, 2021 | Cross-sectional | Canada | Adults and children ≥ 1 y | WG intake  Low, mid, and high intake based on age-specific tertiles of whole grain intake | No WG intake | - WG intake positively associated with fiber, vitamin B6 (children only), thiamin (adults only), potassium, zinc (adults only), calcium, iron, magnesium, folate, decreased total fat & sat fat (adults only) - WG intake not associated with sugar or sodium intake - Top 2 sources of WG were whole grain oat/high fiber breakfast cereal and whole grain/whole wheat bread | energy intake, gender, age, BMI category, income, and supplement use |
| Vatanparast, 2019 | Cross-sectional | Canada | Adults and children ≥ 2 y | RTEC consumers | RTEC non-consumers | - 22% of population consumed RTEC on any given day - 38% of children 2 – 12 y, 29% of adolescents 13 – 18 y, and 19% of adults ≥ 19 y consumed RTEC on any given day - RTEC consumers had higher intakes of nutrients to encourage compared to RTEC non-consumers - More than 15% of daily intake of folic acid, iron, thiamin, and vitamin B6 were contributed by RTEC | ethnicity, education, smoking, food security, age, immigration, and energy intake |
| Zhou, 2023 | Cross-sectional | USA | Adults ≥ 19 y | fortification of grain foods with folate | NA | - Adults getting folate from dietary sources, including RTEC, (no supplements) did not exceed the upper limit of intake - Adults consuming folate from natural sources alone did not meet EAR, but when fortified foods were added the median intake was above the EAR | sex, age category, race/ethnicity, current smoking status, and BMI category |
| Zhu, 2022 | Cross-sectional | USA | Adults and children ≥ 2 y | RTEC breakfast consumers | non-RTEC breakfast consumers  breakfast skippers | - 28% of children and 12% of adults consumed RTEC on any given day - RTEC consumers had higher intakes of carbohydrate, fiber, calcium, magnesium, iron, zinc, phosphorus, potassium, B vitamins, Vitamins A and D - RTEC consumers had similar breakfast intake of sodium, sat fat, added sugar (children only) - Adult RTEC consumers had lower added sugar intake at breakfast compared to breakfast skippers - RTEC consumers were more likely to meet EAR for nutrients | energy intake, age, gender, race/ethnicity, and ratio of family income to poverty |
| Zhu, 2019 | Cross-sectional | USA | Adults ≥ 19 y | RTEC consumers | RTEC non-consumers | - 19% of adults were RTEC consumers - RTEC consumers had similar levels of energy intake and non-consumers - RTEC consumers had higher intakes of dietary fiber, calcium, iron, magnesium, potassium, zinc, vitamin A, thiamin, riboflavin, niacin, vitamin B6, folate, vitamin B12, and vitamin D - RTEC consumers were more likely to meet nutrient recommendations - RTEC consumers had the same added sugar intake as non-consumers | energy intake, age, race/ethnicity, income to poverty ratio, education, smoking status |
| **Food group intake/Diet quality** | | | | | | | |
| Ak, 2015 | Cross-sectional | Malaysia | Children and adolescents  6 – 17 y | sources of WG | NA | - RTEC was the major source of whole grain intake for children and adolescents (more than 60%) | none |
| Albertson, 2016 | Cross-sectional | USA | Children 6 – 18 y and Adults 19+ y | sources of WG | NA | - RTEC is a significant source of WG in the diet for children, but has declined from 2001/2 to 2011/12 (39.6% to 27.1%) - RTEC is also an important source of WG in the diet for adults, and has also been trending down (25.4% to 18.6%). | age, age², gender, race/ethnicity, total calorie intake (kcal), alcohol intake, and physical activity |
| Fayet-Moore, 2019 | Cross-sectional | Australia | Adults  >19 y | RTEC for breakfast | Skipping  Non-cereal breakfast | - RTEC consumers had healthier diets compared to non-RTEC and skipper (lower added sugar, lower total and sat fat; higher CHO & fiber and all micronutrients except Na) (fiber due to higher intake at bf) - RTEC consumers most likely to meet recommendations for grain, fruit, dairy and vegetable and had highest WG intake (double non-cereal bf and triple bf skipper) - RTEC consumers had lowest intake of refined grains; non-cereal breakfast had twice as much refined grain foods and skippers had 3 times refined grain - RTEC consumers more likely to consume fruit and dairy at breakfast – double that of non-cereal bf | sex, age group, their interaction, BMI group, and energy intake at total day, breakfast, or the rest of the day |
| Fulgoni, 2019 | Cross-sectional | USA | Children & adolescents  2 – 18 y | Low sugar ≤ 21.2% and high sugar >21.2% RTEC breakfast | Oatmeal | - RTEC consumers have higher intakes of iron and folate compared to oatmeal, but lower intake of WG, magnesium, and fiber (magnesium and fiber only different from high sugar RTEC) | age, gender, race/ethnicity, poverty income ratio, physical activity level, and kcal |
| Hassan, 2020 | Cross-sectional | Australia | Children 1 – 2 years | sources of WG | NA | - RTEC was a major food source of WG (40%) in these children | NA |
| Lyons, 2018 | Cross-sectional | Ireland | Adults  18 – 64 y | Portion sizes of RTEC and energy density  Tertiles of portion sizes were 30 g, 45 g, and 72 g | NA | - Energy density was higher on days when larger portions of RTEC were consumed | energy intake |
| McGill, 2015 | Cross-sectional | USA | Adults and children  4 – 18 y  19 – 50 y  ≥ 51 y | Food sources of WG and fiber | NA | - RTEC was a major contributor of WG (31% of WG was from RTEC) in children/ adolescents and adults (23% of WG from RTEC) - No improvements in WG or fiber intake from RTEC from 2001 - 2010 | age, gender, race-ethnicity, socioeconomic status, and education |
| McGill, 2016 | Cross-sectional | USA | Adults 19+y | Food sources of fiber | NA | - People meeting AI for fiber had higher HEI score | age, gender, and ethnicity |
| Michels, 2016 | Cross-sectional | Europe | Adolescents  12.5 – 17.5 y | RTEC breakfast | Bread breakfast  Other non-RTEC breakfast | - RTEC consumers had higher frequency of fruit and dairy intake with breakfast | gender, age, socio-economic status, and city |
| Neo, 2016 | Cross-sectional | Singapore | Children 6 – 12 y | sources of WG | NA | - RTEC contributed 8.2% of daily WG intake | none |
| O’Connor, 2015 | Cross-sectional | Ireland | Children and adults 5 – 90 y | determinants of dietary energy density | NA | - Individuals in the highest tertile of RTEC consumption had higher dietary energy density compared to medium and low tertile of RTEC consumption | age |
| O’Donovan, 2019 | Cross-sectional | Ireland | Adults 18 – 90 y | sources of WG | NA | - RTEC was the second largest contributor to WG intake (26%) and the largest contributor at breakfast (40%) | age group, social class, and eating location |
| O’Neil, 2015 | Cross-sectional | USA | Children  2 – 18 y | RTEC consumption patterns (including presweetened RTEC and whole milk, presweetened RTEC and low fat milk, RTEC and whole milk, RTEC and low fat milk) | Skipping breakfast | - Patterns containing RTEC (presweetened and non-presweetened) with low fat milk had higher HEI-2005 scores than skippers | self-reported age, gender, race/ethnicity, and poverty income ratio (PIR) grouped into three categories (< 1.25, 1.25–3.49, and > 3.49), physical activity, energy intake |
| Poinsot, 2024 | Validation study | France | Children 4 – 17 y | assessment of breakfast quality score | NA | - RTEC breakfasts had the highest breakfast quality score (74/100 for children, 73/100 for adolescents) | NA |
| Rehm, 2017 | Modeling | USA | Adults & children  ≥ 1 y | Replacement of breakfast foods with RTEC on per calorie basis  (RTEC alone and RTEC + milk) | Observed breakfast | - Replacement with RTEC alone increased WG intake - Replacement with RTEC + milk increased WG and dairy food group. - Diet quality higher with replacement with RTEC + milk | recall data (weekday/weekend), recall order (first/second), and mode (telephone/in-person) |
| Sanders, 2023 | Cross sectional | Canada | Adults and children  2 – 18 y  19+ y | RTEC consumption by income level | No RTEC consumption | - RTEC consumers had higher diet quality - RTEC provided more than 1/3 daily whole grains. | age, sex, and energy intake |
| Smith, 2022 | Cross sectional | USA | Adults and children  2 – 18 y  ≥ 19 y | RTEC consumption by income level | No RTEC consumption | - RTEC consumers had a higher intake of total dairy, fluid milk, total fruit (adults only), intact fruit (adults only) and WG. - RTEC consumers also had lower intake for children and adults of refined grains (children only), total protein foods, and total meat, poultry, seafood, and egg intake - RTEC consumption was significantly associated with higher diet quality for children and adults. - RTEC contributed to one third or more of daily intake, across all ages for WG. | age, energy intake, gender, and race/ethnicity |
| Smith, 2021 | Cross-sectional | Canada | Adults and children ≥ 1 yr | WG intake  Low, mid, and high intake based on age-specific tertiles of whole grain intake | Less/no WG intake | - WG intake positively associated with overall diet quality, fruit (adults only), legumes, nuts & seeds (adults only), refined grains, meat and poultry (adults) - Top 2 sources of WG were whole grain oat/high fiber breakfast cereal and whole grain/whole wheat bread | energy intake, gender, age, BMI category, income, and supplement use |
| Vatanparast, 2019 | Cross-sectional | Canada | Adults and children ≥ 2 y | RTEC consumers | RTEC non-consumers | - 66% of milk consumption was co-consumed with RTEC in RTEC consumers - Nutrient density of overall diet higher in RTEC consumers compared to non-consumers | ethnicity, education, smoking, food security, age, immigration, and energy intake |
| Zhu, 2022 | Cross-sectional | USA | Adults and children ≥ 2 y | RTEC breakfast consumers | non-RTEC breakfast consumers  breakfast skippers | - RTEC consumers had higher intakes of whole grains and dairy - RTEC consumers had higher diet quality | energy intake, age, gender, race/ethnicity, and ratio of family income to poverty |
| Zhu, 2019 | Cross-sectional | USA | Adults ≥ 19 y | RTEC consumers | RTEC non-consumers | - RTEC consumers had higher intakes of dairy, fluid milk, total fruits (particularly >55 y), whole fruit intake, and WG intake, but lower intakes of protein foods - RTEC consumers had better diet quality than non-consumers (HEI 57.6 vs 50.1) | energy intake, age, race/ethnicity, income to poverty ratio, education, smoking status |
| **Affordability** | | | | | | | |
| Brauchla, 2022 | Cross-sectional | USA | Adults 19+ y | Cost-effective sources of nutrients and food groups | NA | - RTEC (regardless of sugar content) was one of the top 10 sources of WG in the diet (low sugar rank 6, high sugar rank 8) - RTEC (regardless of sugar content) was one of the top 10 sources of iron, Zn, and vitamin A (top source for zinc) - RTEC provide 14.8% of daily iron intake and 7.6% daily Zn intake - For each dollar spent, RTEC provides > 2 oz equivalents of WG | adjusted for the complex sample design of NHANES using appropriate survey weights, strata, primary sampling units, and day one dietary sample weights |
| Rehm, 2017 | Modeling | USA | Adults & children  ≥ 1 y | Replacement of breakfast foods with RTEC on per calorie basis  (RTEC alone and RTEC + milk) | Observed breakfast | - Diet cost lower with replacement with RTEC + milk | recall data (weekday/weekend), recall order (first/second), and mode (telephone/in-person) |
| Sanders, 2023 | Cross sectional | Canada | Adults and children  2 – 18 y  19+ y | RTEC consumption |  | - RTEC consumers had higher diet quality and nutrient intake across all income levels. | age, sex, and energy intake |
| Zhu, 2022 | Cross-sectional | USA | Adults and children ≥ 2 y | RTEC breakfast consumers | non-RTEC breakfast consumers  breakfast skippers | - For children, breakfast meal costs were less for RTEC breakfast than non-RTEC breakfast but total daily meal costs were similar for all RTEC consumers and non-RTEC breakfast consumers | energy intake, age, gender, race/ethnicity, and ratio of family income to poverty |
| **Obesity/BW** | | | | | | | |
| Barr, 2016 | Cross-sectional | Canada | Adults 18+ y | RTEC breakfast consumers | Non-RTEC breakfast consumers  Breakfast skippers | - BMI was significantly lower among RTEC-breakfast consumers compared to other breakfasts (0.6 kg), but not breakfast skippers - The odds of overweight/obesity is not different based on breakfast consumption or type of breakfast. | age, sex, race, household food security, marital status, language spoken at home, physical activity category, smoking, education level, and supplement use |
| Barrientos-Gutierrez, 2023 | Longitudinal cohort | Mexico | Children  5 – 11 y from prenatal birth cohort | RTEC consumption | No RTEC consumption | - There is a marginally significant association of RTEC intake with reduced BMI (-0.098 kg/m2 per 100 kcal/d) | energy intake, children's age and sex, mother's age, education, marital status, and BMI, change in physical activity (min/day), and change in TV watching (min/day), and BMI |
| Castillo Valenzuela, 2015 | Cross-sectional | Chile | Children  6 – 13 y | RTEC consumption  1-29 g/d (1 portion or less), 30-59 g/d (1 to 2 portions), and ≥60 g/d (2 or more portions) | Lower or no RTEC consumption | - RTEC consumption was associated with reduced BMI and WC - RTEC consumption associated with lower risk of overweight/obesity OR:0.53 (0.32 – 0.89) in highest vs no consumption | gender, age, pubertal maturation, physical inactivity (hours per day in front of a screen), and socioeconomic level |
| Chung, 2015 | Cross-sectional | Korea | Children and adults  1 – 75 y | RTEC consumption | Non-RTEC consumption | - RTEC consumers did not differ in BMI from non-consumers - There was no difference in odds of obesity, diabetes, or metabolic syndrome between RTEC consumers and non-consumers. | gender, age group, education, residential area, and occupation |
| Fayet-Moore, 2019 | Cross-sectional | Australia | Adults  >19 y | RTEC for breakfast | Skipping  Non-RTEC breakfast | - RTEC consumption increased with age - RTEC consumers had lowest BMI and WC compared to non-RTEC and skipper | sex, age group, their interaction, BMI group, and energy intake at total day, breakfast, or the rest of the day |
| Fayet-Moore, 2016 | Cross-sectional | Australia | Children  2 – 16 y | RTEC for breakfast | Skipping  non-RTEC bf | Lower prevalence of OW in RTEC vs non-RTEC, but no diff in BMI or WC | age, sex, and physical activity level |
| Fayet-Moore, 2017 | Cross-sectional | Australia | Children & adolescents  2 – 18 y | RTEC breakfast consumers  minimally pre-sweetened(MPS) < 15% or pre-sweetened (PS) >15% or PS>30% | Non-cereal breakfast consumers  skippers | - No difference in prevalence of ow/ob or BMI z-score | age, sex, BMI z-score, and energy intake |
| Keogh, 2020 | RCT | Australia | Adults with ow/ob 18+ y | RTEC breakfast 5d/wk + wt loss diet | 2 egg breakfast 5 d/wk + wt loss diet | - Both treatments lost weight and no difference between the treatments | NA |
| Kuriyan, 2017 | RCT | India | Adult women with ow/ob  18 – 44 y | Low-fat RTEC breakfast and lunch for 2 wk | Dietary advice for wt loss | - RTEC treatment lost more weight had a greater change in BMI, and greater reductions in the waist, abdominal waist, and hip circumference than the comparator - No effect on appetite - intakes of niacin, vitamin B-6, B-12, fiber and sugar were significantly greater in the intervention group | NA |
| Michels, 2015 | Cross-sectional | Europe | Adolescents  12.5 – 17.5 y | RTEC consumption frequency  (once/wk, 2–4 times/wk, ≥ 5-6 times/wk) | No-RTEC consumption | - RTEC consumers were 57% less likely to be overweight - Fasting glucose and lipids were not different | sex, age, socio-economic status, city, and breakfast skipping |
| O’Neil, 2015 | Cross-sectional | USA | Children  2 – 18 y | RTEC consumption patterns (including presweetened RTEC and whole milk, presweetened RTEC and low fat milk, RTEC and whole milk, RTEC and low fat milk) | Skipping breakfast | - Presweetened and non-presweetened RTEC with whole milk had lower BMI z-score compared to skippers (no diff with RTEC + low fat milk) | self-reported age, gender, race/ethnicity, and poverty income ratio (PIR) grouped into three categories (< 1.25, 1.25–3.49, and > 3.49), physical activity, energy intake |
| Quatela, 2017 | Longitudinal cohort (12 y follow up) | Australia | Middle-aged women  Mean 52.4 y  45 – 50 y at initiation of cohort | RTEC consumers  Also specific categories (All-Bran, Sultana Bran/Fibre Plus/Branflakes, Weet Bix/Vita Brits/Weeties, Cornflakes/Nutrigrain/Special K, muesli, oat-based, wheat based, higher fiber or whole grain | RTEC non-consumers | - RTEC consumption was not associated with risk of obesity. OR = 0.92 (0.63 – 1.35) - All-Bran, oat cereal or muesli consumption was associated with reduced risk of obesity (OR 0.62, 0.71 or 0.57, respectively) | residency, income, smoking, physical activity, hypertension, daily energy intake, daily fibre intake, and other breakfast cereal consumption |
| Shaw, 2015 | RCT | Ireland | Healthy but ow/ob men and women  20 – 60 y | Special K cereal for breakfast and lunch for 2 wk | Normal diet | - Special K challenge reduced daily energy intake by ~673 kcals/d - BMI, body fat mass, waist circumference, and lean tissue mass were significantly reduced with the Special K Challenge | NA |
| Shyam, 2019 | Cross-sectional | Malaysia | Adults  18 – 60 y | Dietary pattern including RTEC (also included bread, legumes, spreads, vegetables, milk and milk products, malted chocolate drinks, soy milk, corn, and confectionery | Compared quartiles of adherence to pattern | - No association of RTEC dietary pattern with overweight or obesity | age, sex, ethnicity, household income |
| Vatanparast, 2019 | Cross-sectional | Canada | Adults and children ≥ 2 y | RTEC consumers | RTEC non-consumers | - RTEC consumption was not associated with overweight/obesity | ethnicity, education, smoking, food security, age, immigration, and energy intake |
| LaFond, 2015 | RCT | USA | Adult women with overweight  18 – 29 y | High-fiber RTEC (low molecular weight soluble fiber) | Low-fiber RTEC | - No difference in postprandial appetite after both | NA |
| **Type 2 Diabetes** | | | | | | | |
| Chen, 2023 | Three prospective cohorts | USA | Adults  25 – 75 y | Cold cereals (as a form of UPF) | Other types of UPF | - Intake of RTEC associated with lower type 2 diabetes risk. Dietary fiber and mineral content mediated some of the association. | age, race/ethnicity, family history of T2D, baseline history of hypertension and/or hypercholesterolemia, smoking status, physical activity level, alcohol consumption, menopausal status, postmenopausal hormone use, oral contraceptive use, history of physical examination, neighborhood income, and total energy intake |
| Dicken, 2024 | Prospective Cohort | Europe (8 countries – EPIC) | Adults  35 – 70 y | Cold cereals (as a form of UPF) | Other types of UPF | - Bread, biscuit, and breakfast cereal consumption associated with lower type 2 diabetes risk (HR: 0.65; CI:0.57 – 0.73) | study center, self-reported sex (male, female), highest education level (none, primary school, technical/professional, secondary, higher education, not specified/missing), occupation (employed, housewife, retired, unemployed, student, other, missing), history of previous illness (cardiovascular disease, cancer, hypertension, hyperlipidaemia), family history of type 2 diabetes mellitus in a parent or sibling (yes, no, missing), smoking status and intensity (never, current 1–15 cigarettes/day, current 16–25 cigarettes/day, current 26+ cigarettes/day, quit £10 years, quit 11–20 years, quit >20 years, current pipe/cigar/occasional smoker, current/ former with missing intensity, missing), physical activity level (inactive, moderately inactive, moderately active, active, missing), alcohol intake (g/day), menopausal status (premenopausal, postmenopausal, perimenopausal, surgical postmenopausal), and use of hormone replacement therapy (HRT) or oral contraceptives (yes, no). |
| Hu, 2020 | Prospective Cohort | USA | Adults  25 – 75 y | WG RTEC intake | less or no WG RTEC intake | - Individuals consuming one or more svgs/d of whole grain RTEC had a lower risk of type 2 diabetes compared to those consuming less than a svg/month (HR: 0.81, 0.77 – 0.86) | age (years), ethnicity (white, African American, Asian, others), smoking status (never smoked, past smoker, currently smoke 1-14 cigarettes/day, 15-24 cigarettes/day, or ≥25 cigarettes/day), alcohol intake (0, 0.1-4.9, 5.0-9.9, 10.0-14.9, 15.0-29.9, and ≥30.0 g/day), multivitamin use (yes, no), physical activity (divided into five equal groups), modified alternative healthy eating index (whole grain removed, divided into five equal groups), and family history of diabetes. For women, postmenopausal hormone use (never, former, or current hormone use, or missing), and oral contraceptive use, time varying body mass index (<21.0, 21.0-22.9, 23.0-24.9, 25.0-26.9, 27.0-29.9, 30.0-32.9, 33.0-34.9, or ≥35.0) |
| **Cardiovascular Health** | | | | | | | |
| Chung, 2015 | Cross-sectional | Korea | Adults  19 – 75 y | RTEC consumption | Non-RTEC consumption | - RTEC consumption was inversely associated with blood pressure, serum triglyceride and total cholesterol - Prevalence of hypertension was significantly lower in RTEC consumers compared to non-consumers OR 0.19 (0.06, 0.60) | gender, age group, education, residential area, and occupation |
| Hu, 2022 | Prospective cohort | USA | Adults  25 – 75 y | WG RTEC intake | less or no WG RTEC intake | - Individuals consuming one or more svgs/d of whole grain RTEC had a lower risk of coronary heart disease compared to those consuming less than a svg/month (HR: 0.83, 0.78 – 0.89) | age (years), ethnicity (white, African American, Asian, others), updated body mass index (<21.0, 21.0–22.9, 23.0–24.9, 25.0–26.9, 27.0–29.9, 30.0–32.9, 33.0–34.9, or ≥35.0 kg/m2), smoking status (never smoked, past smoker, currently smoke 1–14 cigarettes per day, 15–24 cigarettes per day, or ≥25 cigarettes per day), alcohol intake (0, 0.1–4.9, 5.0–9.9, 10.0–14.9, 15.0–29.9, and ≥30.0 g/day), baseline diabetes (yes, no), multivitamin use (yes, no), physical activity (quintiles), modified alternative healthy eating index (quintiles, whole grain component was excluded), total energy (quintiles), and family history of MI (yes, no). For women, postmenopausal hormone use (never, former, or current hormone use, or missing) and oral contraceptive use (yes, no) |
| Juan, 2017 | Prospective cohort | USA | Adults  25 – 75 y | WG RTEC intake | Less or no WG RTEC intake | - Intake of WG breakfast cereal (> 1 svg/wk) was inversely associated with stroke. HR: 0.88 (0.80 – 0.96) - Intake of bran was inversely associated with stroke. HR: 0.89 (0.79 – 1.00) - Intake of total WG was not associated with stroke. HR: 1.04 (0.91 - 1.19) nor were other WG foods (brown rice, popcorn, dark bread, oatmeal | age, ethnicity, body mass index (BMI), smoking status, alcohol intake, physical activity, multivitamin use, family history of myocardial infarction (MI), cancer, or diabetes, hypertension, high cholesterol at baseline, total energy intake, and the modified alternative healthy eating index (whole grain removed) score, for women - menopausal status and postmenopausal hormone use |
| Mendoza, 2024 | 3 Prospective cohorts | USA | Adults  Cohort 1: 30 – 55 y female at recruitment  Cohort 2: 25 – 42 y female at recruitment  Cohort 3: 40 – 75 y male at recruitment | Cold cereals (as a form of UPF) | Other types of UPF | - RTEC, even though considered ultra-processed, is inversely associated with cardiovascular disease risk - RTEC intake associated with lower risk of CVD, CHD, and stroke | race/ethnicity, marital status, working status, smoking status, quintiles of physical activity (METhours/week), sleep patterns (hours/day), family history of CVD, multivitamin use, aspirin use, NSAID use, menopausal hormone use status (women only), oral contraceptive use (women only), energy intake, BMI at baseline, hypertension at baseline, hypercholesterolemia at baseline, and diabetes at baseline. |
| Ponce-Martinez, 2018 | Cross-sectional | Mexico | Adults  20 – 50 y | High-fiber or refined grain RTEC consumption  (≥once/week) | Low fiber or no RTEC consumption  (≤3 times/month or never ) | - Consumption of 1 bowl of high-fiber breakfast cereal more than once per week was associated with a decreased risk of having high blood pressure when compared to the reference category (≤3 times per month or never). OR = 0.73 (0.53 – 0.98) - The ≥once/week group had higher sodium intake than the ≤3 times per month or never - Consumption of 1 bowl of refined grain breakfast cereal was not associated with risk of elevated blood pressure | age, sex, education level, BMI, alcohol consumption, tobacco use, energy (kcal/day), potassium (mg/day), magnesium (mg/day), and frequency of intake of bacon, ham, sausages, and cheese |
| Setayeshgar, 2015 | Cross-sectional | Canada | Adults  30 – 74 y | RTEC consumption  (<4 times/wk, ≥4 times/wk) | Low or no RTEC consumption  (<1 times/wk) | - Intake ≥ 4 times/wk significantly decreases odds of having increased risk of CVD | age, sex, education, income, ethnicity, physical activity, education, household income, abdominal obesity and drinking alcohol |
| **Mortality** | | | | | | | |
| Lin, 2025 | Prospective cohort | UK | Adults  40 – 69 y | Breakfast cereal consumption and type  Muesli, plain, bran, whole wheat cereal (0 bowl/d, > 0-0.5 bowl/day, > 0.5-1 bowl/day, and > 1 bowl/day)  Similar for oat, sweetened, and other cereal except no >1 bowl/d category due to low subject numbers | No breakfast cereal consumption | - Overall breakfast cereal consumption correlated to lower all-cause and CVD-related mortality, but did not reach significance for cancer - Muesli, bran and oat cereal intake significantly correlated to lower all-cause mortality at moderate consumption levels (up to 1 bowl/d but not higher) - Muesli, bran and “other” cereal types significantly correlated to lower CVD mortality at moderate consumption levels (up to 1 bowl/d but not higher) - Moderate intake of plain cereal correlated to increased risk of CVD mortality - Moderate intake of muesli and bran (0.5 – 1 bowl/d) was associated with reduced risk of cancer mortality - Sweetened cereal intake (>0.5 bowls/d) was associated with increased risk of cancer mortality - Adding dried fruit to any breakfast cereal was associated with lower all-cause, CVD and cancer mortality - Adding sugars or artificial sweeteners attenuated the inverse association of breakfast cereal with all-cause, CVD and cancer mortality. - Adding milk to breakfast cereal did not influence the association of breakfast cereal with all-cause mortality | age, sex, ethnicity, Townsend deprivation index, education, body mass index, smoking status, alcohol consumption, physical activity, dietary intake of various nutrients and foods (red meat, vegetables, fruit, starchy food, bread, milk, coffee, tea, total intake of energy, fat, and sugar), supplements of vitamin and minerals, medical history (hypertension, diabetes, high cholesterol, long-standing illness, the use of cholesterol drug, hypertension drug, and insulin drug, family history of CVD and cancer) |
| Xu, 2016 | Prospective cohort | USA | Adults  50 – 71 y | RTEC consumption  (quartiles of grams/d) | Low or no RTEC consumption | - Highest quartile of RTEC consumption associated with reduced risk of all-cause mortality [0.85 (0.83, 0.88)], CVD mortality [0.76 (0.71, 0.81)], cancer mortality [0.90 (0.86, 0.95), and digestive cancer mortality [0.87 (0.79, 0.97)] compared to non-consumers - Within RTEC consumers, those with the highest fiber intake had reduced risk of all-cause mortality [ 0.86 (0.82, 0.89)], CVD mortality [0.88 (0.86, 0.91)], cancer mortality [0.90 (0.85, 0.96)], and digestive cancer mortality [0.83 (0.73, 0.94)]. | age, gender, smoking status, smoking dose, and time since quitting smoking, race/ethnicity, education, marital status, self-rated health status, body mass index, physical activity, menopausal hormone therapy use, and intake of alcohol, red meat, fruits, vegetables, and total energy |
| Zhang, 2024 | Prospective cohort | UK | Adults  mean age 56 y (range not provided) | Sweetened, unsweetened and artificially sweetened RTEC intake  (based on what is added by consumer)  (>0 to 0.5 bowls, >0.5 to 1.5 bowls, and >1.5 bowls / day | RTEC non-consumers  (0 bowls/d) | - Consumers of unsweetened RTEC had lower risk of all-cause mortality compared to non-consumers. 0 – 0.5 bowls/d = 0.89 (95%CI: 0.84–0.95), 0.5 – 1.5 bowls/d = 0.90 (95%CI: 0.86–0.94), and >1.5 bowls/d = 0.89 (95%CI: 0.82–0.97). - Consumers of unsweetened RTEC had lower risk of CVD mortality compared to non-consumers. 0 – 0.5 bowls/d = 0.82 (0.69–0.98), 0.5 – 1.5 bowls/d = 0.86 (0.75–0.98). - Unsweetened RTEC intake was not associated with cancer mortality - Sweetened cereal and artificially sweetened cereal intake was not associated with risk of all-cause or CVD mortality | age, sex, BMI, basal metabolic rate, ethnicity, Townsend deprivation index (TDI), household income, education, smoking status, pack-years of smoking, physical activity level, vitamin use, mineral and other dietary supplement use, NSAID use, healthy sleep pattern, family history of CVD, family history of cancer, number of long-term conditions, intake of total energy, total sugar, coffee, starchy food, refined grains, snacks, pizza, and modified AHEI |
